# In vitro toxicokinetics and metabolic profiling of methoxycathinones and methylthiocathinones using human liver systems and hyphenated mass spectrometry

**DOI:** 10.1007/s00204-025-04205-x

**Published:** 2025-09-29

**Authors:** Matthias D. Kroesen, Tanja M. Gampfer, Lea Wagmann, Pierce V. Kavanagh, Simon D. Brandt, Markus R. Meyer

**Affiliations:** 1 Department of Experimental and Clinical Toxicology and Pharmacology, Center for Molecular Signaling (PZMS), PharmaScienceHub (PSH), Saarland University, Homburg, Germany; 2 Department of Pharmacology and Therapeutics, School of Medicine, Trinity Centre for Health Sciences, St. James Hospital, Dublin 8, Dublin, Ireland; 3 The Alexander Shulgin Research Institute, 1483 Shulgin Road, Lafayette, CA 94549 USA

**Keywords:** HepaRG, New psychoactive substances, Synthetic cathinones, Analytical toxicology

## Abstract

Ring-substituted synthetic cathinones represent a major subgroup within new psychoactive substances. This study investigated the in vitro toxicokinetics of the three 4-methoxy-substituted representatives 4MeO-NE-BP (4’-methoxy-*N*-ethylbutyrophenone), 4MeO-αP-BP (4’-methoxy-α-pyrrolidinobutyrophenone), and 4MeO-αP-VP (4’-methoxy-α-pyrrolidinovalerophenone) and the three related novel 4-methylthio analogs 4MeS-NE-BP (4’-methylthio-*N*-ethylbutyrophenone), 4MeS-αP-BP (4’-methylthio-α-pyrrolidinobutyrophenone), and 4MeS-αMor-PrP (4’-methylthio-2-morpholinopropiophenone). This included plasma protein binding (PPB), phase I and phase II metabolism in pooled human liver S9 fraction (pHLS9) and HepaRG cells, and monooxygenases activity. Methoxycathinones exhibited lower PPB (~ 40–60%) compared to methylthiocathinones (~ 85%). Predominant phase I metabolic reactions included *O*-/*S*-demethylation and hydroxylation, with additional transformations such as *N*-dealkylation, *N*-oxidation, and oxo reduction. Phase II conjugation reactions, such as glucuronidation and sulfation, were observed post-demethylation. Overall, 42 and 45 metabolites were identified in pHLS9 and HepaRG systems, respectively, with metabolite number increasing alongside alkyl chain length and heterocyclic substitution. All compounds were substrates for multiple monooxygenases, suggesting a low risk for drug–drug interactions. Based on metabolic stability and abundance, parent compounds and *O*-/*S*-desmethyl and hydroxylated metabolites might be proposed as urinary screening targets in clinical and forensic toxicology, as well as doping control settings.

**Supplementary Information:**

The online version contains supplementary material available at 10.1007/s00204-025-04205-x.

## Introduction

Drug abuse and drug-induced deaths involving synthetic stimulants continue to cause concern (EUDA 2024). In 2022, stimulants represented the second largest category of new psychoactive substances (NPS) monitored by the EU Early Warning System (EUDA 2022). The use of synthetic cathinones has increased sharply in recent years. Among stimulant users who did not primarily consume cocaine, 4% reported using synthetic cathinones as a gateway to stimulant abuse in 2016, and by 2022, this proportion had doubled to 8% (EUDA 2024). In general, NPS are introduced to the market without prior safety testing, and data on their toxicokinetics and toxicodynamics remain limited or entirely absent.

**Fig. 1:**
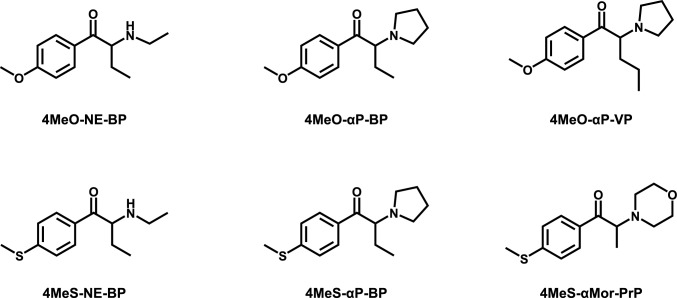
Chemical structures of the synthetic cathinones 4MeO-NE-BP, 4MeO-αP-BP, 4MeO-αP-VP, 4MeS-NE-BP, 4MeS-αP-BP, and 4MeS-αMor-PrP

Many synthetic cathinones are characterized by ring substitution, with one particular subgroup featuring the 4’-methoxy group, such as methedrone (4’-methoxymethcathinone, 4MeO-MC). At the same time, data on sulfur-containing analogs appear to be less common or unavailable. The present study investigated three less common 4’-methoxy-substituted cathinones, namely 4MeO-NE-BP (4’-methoxy-*N*-ethylbutyrophenone, 4’-methoxy-*N*-ethylbuphedrone), 4MeO-αP-BP (4’-methoxy-α-pyrrolidinobutyrophenone, also known as 4-MeOPBP or 4-methoxy-α-PBP), and 4MeO-αP-VP (4’-methoxy-α-pyrrolidinovalerophenone, also known as 4-MeOPVP or 4-methoxy-α-PVP), and three novel 4’-methylthio analogs 4MeS-NE-BP (4’-methylthio-*N*-ethylbutyrophenone, 4’-methylthio-*N*-ethylbuphedrone), 4MeS-αP-BP (4’-methylthio-α-pyrrolidinobutyrophenone), and 4MeS-αMor-PrP (4’-methylthio-2-morpholinopropiophenone). Compound names follow a systematic nomenclature framework (Pulver et al. 2024) and their structures are shown in Fig. 1.

Pharmacological data are available for some of the compounds or their closely related analogs. For example, 4MeO-αP-VP has been reported to inhibit monoamine uptake, showing greater potency in dopamine than serotonin transporters (Wojcieszak et al. 2021). It has been seized multiple times and was detected in a forensic case, after which the metabolism was determined in vitro (Ellefsen et al. 2016; Mochizuki et al. 2021). A fatality has been reported by 4MeO-αP-BP use, but no metabolism data were reported (Shintani-Ishida et al. 2015). 4MeS-αMor-PrP is a monomethyl analog at the 2-position of the dimethyl compound Irgacure-907 (MMMP), a cathinone derivative which is commercially used as a photoinitiator. Because of its structure, Irgacure-907 is subject to misuse and was already identified in a fatality and as a food contaminant in commercial cow milk (Nash et al. 2019; Tseng et al. 2021). Furthermore, Irgacure-907 was identified by eight customs in Europe and ex-Europe in the USA, Australia, and Brazil (Guillou et al. 2018). Structurally related cathinones such as α-PVP and α-PBP are known to act as catecholamine transporter blockers that induce increased locomotor activity (Marusich et al. 2014). Notably, the *N*-ethyl-substituted derivatives 4MeO-NE-BP and 4MeS-NE-BP and 4MeS-αP-BP have not yet been described in the literature, but it is hypothesized that they may exhibit at least some of these effects.

To date, only limited or no information is available regarding the toxicokinetic properties of the six cathinones and no urinary biomarker are available to serve as screening targets in toxicological analysis and doping control. To generate such comparative toxicokinetic data, study design included assessment of in vitro plasma protein binding (PPB), in vitro metabolism using pooled human liver S9 fractions (pHLS9) and HepaRG cells, and monooxygenase activity screening.

## Materials and methods

All six test drugs (4MeO-NE-BP, 4MeO-αP-BP, 4MeO-αP-VP, 4MeS-NE-BP, 4MeS-αP-BP, and 4MeS-αMor-PrP) were synthesized as hydrochloride salts following standard procedures (Meltzer et al. 2006). Their identity and purity (≥ 95%) were confirmed by mass spectrometric and spectroscopic methods. Stock solutions of each compound were prepared in methanol at a concentration of 1 mg/mL. Before cell incubation, the compounds were prepared and sterile filtered as a 1 mg/mL stock solution in dimethyl sulfoxide (DMSO). 3’-Phosphoadenosine-5’-phosphosulfate (PAPS), DMSO, dipotassium hydrogen phosphate (K_2_HPO_4_), dithiothreitol (DTT), isocitrate, isocitrate dehydrogenase, magnesium chloride (MgCl_2_), potassium dihydrogen phosphate (KH_2_PO_4_), reduced glutathione (GSH), acetyl coenzyme A (AcCoA), S-(5’-adenosyl)-l-methionine (SAM), superoxide dismutase, and two-chambered Centrifree devices were purchased from Merck KGaA (Darmstadt, Germany). Nicotinamide adenine dinucleotide phosphate (NADP^+^) was from Sigma-Aldrich (Steinheim, Germany). UDP-glucuronic acid 25 mM (UGT reaction mixture solution A), 250 mM Tris–HCl, 40 mM MgCl_2_, and 125 μg/mL alamethicin (UGT reaction mixture solution B) were obtained from Corning (Amsterdam, Netherlands). Water was purified with a Millipore (Merck, Darmstadt, Germany) filtration unit, purifying water to a resistance of 18.2 Ω × cm. Pooled human liver S9 fraction (20 mg microsomal protein/mL, 150 donors), baculovirus-infected insect cell microsomes (Supersomes) containing human cDNA-expressed cytochrome P450 (CYP) isoforms CYP1A2, CYP2B6, CYP2C8, CYP2C19, CYP2D6, CYP3A4, CYP3A5 (1 nmol/mL); CYP2A6, CYP2C9, and CYP2E1 (2 nmol/mL), and flavin-containing monooxygenase 3 (FMO3, 5 mg/mL) were purchased from Discovery Life Sciences (Huntsville, LA, USA). All enzyme-containing preparations were thawed at 37 °C after delivery, aliquoted, snap-frozen in liquid nitrogen, and stored at − 80 °C until use. Trimipramine-d_3_ and MDMA-HCl were from LGC (Wesel, Germany). Quetiapine was from AstraZeneca (Hamburg, Germany) and penicillin, streptomycin, GlutaMAX, supplement HPRG670, Williams E medium, and cryopreserved and differentiated HepaRG cells (10^7^ cells/cryovial) were purchased from Life Invitrogen (Darmstadt, Germany). Acetonitrile, methanol, formic acid (LC–MS grade each), ammonium formate (analytical grade), and all other reagents and chemicals (analytical grade) were from VWR (Darmstadt, Germany). Collagen-coated 96-well plates were purchased from Sarstedt (Nümbrecht, Germany).

### Plasma protein binding

PPB was investigated according to a previous study with minor modifications (Kroesen et al. 2025). Fresh and pooled human blood plasma (450 µL) was spiked with 50 µL of a 5 µM methanolic solution of 4MeO-NE-BP, 4MeO-αP-BP, 4MeO-αP-VP, 4MeS-NE-BP, 4MeS-αP-BP, or 4MeS-αMor-PrP. The mixture was incubated for 30 min at 37 °C and with gentle shaking at 200 rpm. Plasma aliquots of 100 µL and 400 µL were transferred to a new reaction tube (Global Approach, GA) and onto two-chambered Centrifree devices from Merck KGaA. The Centrifree devices were centrifuged for 35 min at 1600 × *g* to obtain 100 µL of the ultrafiltrate (UF). UF and GA were precipitated using 100 µL acetonitrile (−20 °C) containing 2.5 µM trimipramine-d_3_ as an internal standard. The mixture was vortexed and centrifuged for 2 min at 18,407 × *g*. A volume of 100 µL of the supernatant was transferred into an autosampler vial and 5 µL was injected onto the liquid chromatography − high-resolution tandem mass spectrometry (LC–HRMS/MS) system. Experiments were performed in quadruplicate. Fraction unbound (*f*_*u*_) and PPB were determined by comparing the area ratios of the compound and trimipramine-d_3_ as an internal standard in the UF and GA using the following equations:

1 $${f}_{u}=\frac{\text{peak area ratio}\left(\frac{{\mathrm{compound}}_{\mathrm{UF}}}{{\mathrm{IS}}_{\mathrm{UF}}}\right)}{\text{peak area ratio}\left(\frac{{\mathrm{compound}}_{\mathrm{GA}}}{{\mathrm{IS}}_{\mathrm{GA}}}\right)}$$

2 $$\text{PPB }[\mathrm{\%}]=\left(1-{f}_{u}\right)*100.$$

Lipophilicity (logD) at pH 7.4 of all compounds was calculated with Marvin version 25.1.79 (Chemaxon, Budapest, Hungary).

### Incubations using pooled human liver S9 fraction

Incubations with pHLS9 were performed in accordance with a previous publication (Kroesen et al. 2025). In short, 25 μg/mL alamethicin (UGT reaction mix B), pHLS9 (2 mg microsomal protein/mL), 2.5 mM isocitrate, 0.8 U/mL isocitrate dehydrogenase, 100 U/mL superoxide dismutase, 0.6 mM NADP^+^, and 2.5 mM Mg^2+^ were preincubated for 10 min at 37 °C. Then, 2.5 mM UDP-glucuronic acid (UGT reaction mix A), 40 µM PAPS, 1.2 mM SAM, 1 mM DTT, and 10 mM GSH were added. The reaction was started by adding the substrate (25 µM each, final), resulting in a final incubation volume of 150 µL. MDMA or quetiapine (25 µM and 250 µM final concentrations) was incubated as positive controls. Negative control samples without enzymes and blank samples without substrates were incubated to identify not metabolically formed compounds and to confirm the absence of interfering compounds. Incubations were performed in triplicate and organic solvent in the incubation mixture was below 1% (Chauret et al. 1998). After an incubation time of 60 and 360 min, aliquots (50 µL) were transferred to a new reaction tube. The reaction was stopped with 30 µL acetonitrile (−20 °C) containing 2.5 µM trimipramine-d_3_ as an internal standard. Samples were vortexed, stored at −20 °C for 30 min, and centrifuged for 2 min at 18,407 × *g*. 70 µL of the supernatant were transferred to autosampler vials and 5 µL was injected onto the LC–HRMS/MS system.

### Incubations using HepaRG cells

Cell incubations were performed in a monolayer assay (Kroesen et al. 2025) and in accordance with the manufacturer’s instructions. Further information about the incubation conditions may be found in the Supplementary Information (SI).

In short, MDMA or quetiapine (25 µM and 250 µM final concentrations) was incubated as positive control. All incubations were performed in triplicate, with each well containing 0.5% (*v/v*) DMSO. Following incubation, 50 µL of the medium supernatant was transferred to a new reaction tube and precipitated with 30 µL of acetonitrile cooled to −20 °C, containing 2.5 µM trimipramine-d3 as an internal standard. A negative control sample without HepaRG cells and a blank sample without substrate were used to identify not metabolically formed compounds and to confirm the absence of interfering compounds. The mixture was vortexed and centrifuged for 2 min at 18,407 × *g*. A volume of 70 µL of the supernatant was transferred into an autosampler vial and 5 µL was injected onto the LC–HRMS/MS system.

### Monooxygenases activity screening

As described elsewhere (Kroesen et al. 2025), each compound (25 µM) was incubated with 2.5 mM isocitrate, 0.8 U/mL isocitrate dehydrogenase, 100 U/mL superoxide dismutase, 0.6 mM NADP^+^, 2.5 mM Mg^2+^, and CYP1A2, CYP2A6, CYP2B6, CYP2C8, CYP2C9, CYP2C19, CYP2D6, CYP2E1, CYP3A4, CYP3A5 (50 pmol/mL each), or FMO3 (0.25 mg protein/mL) for 30 min at 37 °C in a final volume of 50 µL. For incubations with CYP2A6 or CYP2C9, phosphate buffer was replaced with 90 mM Tris buffer, according to the manufacturer’s guideline. Thirty µL of the reaction mix was treated with 20 µL acetonitrile (−20 °C) with 2.5 µM trimipramine-d_3_ as an internal standard. All incubations were done in duplicate and concentrations were fixed. Verapamil (25 µM) was incubated as a positive control. Negative control samples without enzymes and blank samples without substrate were incubated to identify not metabolically formed compounds and to confirm the absence of interfering compounds. The amount of organic solvent in the incubation mixture was below 1% (Chauret et al. 1998). Afterward, the samples were vortexed and centrifuged for 2 min at 18,407 × *g*. A volume of 40 µL of the supernatant was transferred to autosampler vials and 5 µL was injected onto the LC–HRMS/MS system.

### LC–HRMS/MS apparatus and conditions

Apparatus settings and measurement conditions are described in SI.

## Results and discussion

### In vitro plasma protein binding

The interaction of drugs with plasma proteins, primarily albumin and α-1 acid glycoprotein (also known as AGP-1), is referred to as plasma protein binding. Plasma protein binding is an important toxicokinetic parameter that plays a crucial role in influencing systemic availability, distribution, and elimination. Table 1 shows the f_u_, PPB, and calculated logD at pH 7.4 of each compound. 4MeO-NE-BP and 4MeO-αP-BP showed a low PPB (< 50%), while 4MeO-αP-VP and all three methylthio compounds showed a moderate PPB (50–90%). For compounds with high PPB (> 90%), release from the plasma protein considerably increases the unbound fraction as a percentage (McLeod and He 2012). In this case, the fluctuation of the unbound fraction is therefore higher than for drugs with low plasma protein binding. Thus, toxicity may be increased, especially if the compounds have a high affinity for their target receptors. This should be taken into consideration, as is the case with methylthiocathinones with an approximate f_u_ of 0.15. In case of a low to moderate PPB of the methoxycathinones, the systematically available fraction is increased (f_u_ 0.4–0.6) and compounds can directly exert effects or could potentially lead to toxicity.

**Table 1:** Toxicokinetic data of 4MeO-NE-BP, 4MeO-αP-BP, 4MeO-αP-VP, 4MeS-NE-BP, 4MeS-αP-BP, and 4MeS-αMor-PrP including plasma protein binding (PPB), unbound fraction (*f*_*u*_), and calculated lipophilicity at pH 7.4 (logD)

Compound	*f*_u_	PPB, %	logD (pH 7.4), calculated
4MeO-NE-BP	0.61	39%	1.42
4MeO-αP-BP	0.54	46%	2.37
4MeO-αP-VP	0.39	61%	2.71
4MeS-NE-BP	0.13	87%	2.22
4MeS-αP-BP	0.16	84%	3.13
4MeS-αMor-PrP	0.14	86%	2.4

The lipophilicity of a substance often correlates with PPB (Laznicek and Laznickova 1995), (Croom 2012). This most likely explained the proportional increase in PPB of the methoxy compounds with an increasing logD. 4MeS-αMor-PrP had a lower calculated logD (2.4) than 4MeS-αP-BP (3.13), but showed a similar PPB, most likely due to steric and electronic effects. The calculated lipophilicity of all compounds was low to moderate (logD value of 1–3), which is consistent with the moderate in vitro PPB. Aromatic methylthio substitution increased the PPB, probably due to electronic and steric effects. Consequently, the half-life of the methylthio compounds could potentially be increased. To avoid an underestimation of the unbound fraction, the compound adsorption to the membrane needs to be minimized. In previous studies, regenerated cellulose membranes were shown to eliminate the effect of non-specific binding and were therefore used to determine the PPB (Heinze and Holzgrabe 2006), (Lier et al. 2024).

### In vitro metabolism by pHLS9 and HepaRG cells

**Fig. 2:**
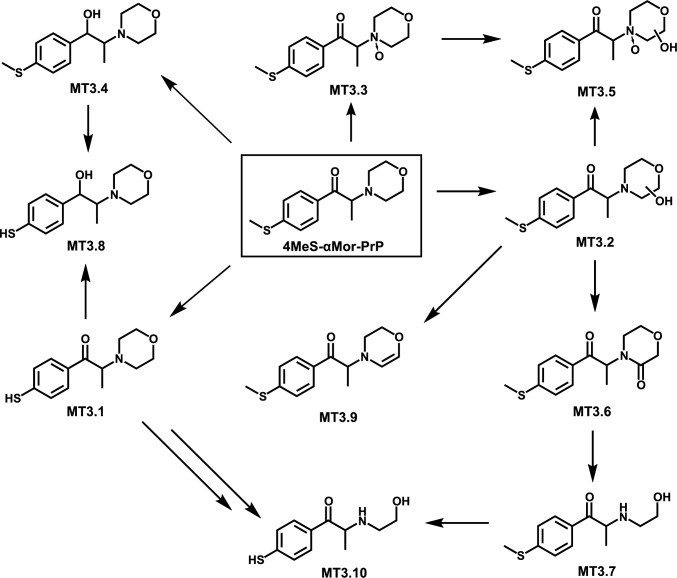
In vitro metabolic pathways of 4MeS-αMor-PrP in HepaRG, pHLS9, and/or monooxygenases incubations. For metabolite IDs, refer to Table S2.6. → , metabolized to

In total, 45 metabolites were tentatively identified. Within the methoxy compound group, 20 metabolites were detected, with 5 metabolites for 4MeO-NE-BP, 5 metabolites for 4MeO-αP-BP, and 10 metabolites for 4MeO-αP-VP. In the methylthio compound group, 25 metabolites were identified, with 7 metabolites for 4MeS-NE-BP, 8 metabolites for 4MeS-αP-BP, and 10 metabolites for 4MeS-αMor-PrP. The metabolic pathways are shown in Figure S1–S5 of SI and, as an example, the pathway of 4MeS-αMor-PrP is illustrated in Fig. 2.

Metabolism of 4MeO-NE-BP included* O*-demethylation (MO1.1), hydroxylation (MO1.2), *N*-dealkylation (MO1.3), hydroxylamine formation (MO1.4), and *O*-demethylation + sulfation (MO1.5). For 4MeO-αP-BP, the metabolic reaction hydroxylation (MO2.1),* O*-demethylation (MO2.2), *N*-oxidation (MO2.3), lactam formation (MO2.4), and hydroxylation + *N*-oxidation (MO2.5) were identified. Metabolic steps of 4MeO-αP-VP were hydroxylation (MO3.1, MO3.3), *O*-demethylation (MO3.2), *N*-oxidation (MO3.4), lactam formation (MO3.5), *O*-demethylation + aromatic hydroxylation (MO3.6), hydroxylation + *N*-oxidation (MO3.7), and *O*-demethylation + dihydroxylation at the pyrrolidine ring (MO3.8). Phase II metabolism included* O*-sulfation (MO3.9) and *O*-glucuronidation (MO3.10) after *O*-demethylation.

Within the group of methylthio-substituted compounds, 4MeS-NE-BP was metabolized via *S*-demethylation (MT1.1), hydroxylation (MT1.2), *N*-dealkylation (MT1.3), hydroxylamine formation (MT1.4), hydroxylation + oxidation (MT1.5), *N*-dealkylation + *S*-demethylation (MT1.6), and hydroxylamine formation + hydroxylation (MT1.7). Metabolic reactions of 4MeS-αP-BP were* S*-demethylation (MT2.1), hydroxylation (MT2.2), *N*-oxidation (MT2.3), lactam formation (MT2.4), *N,N*-bis-dealkylation (MT2.5), dihydroxylation (MT2.6), *S*-demethylation + hydroxylation (MT2.7), and *N*-oxidation + hydroxylation (MT2.8). 4MeS-αMor-PrP was metabolized by* S*-demethylation (MT3.1), hydroxylation (MT3.2), *N*-oxidation (MT3.3), oxo reduction (MT3.4), lactam formation (MT3.6), *N*,*O*-bis-dealkylation (MT3.7), dehydrogenation (MT3.9), and combinations thereof.

More metabolites were found for the heterocyclic compounds such as 4MeS-αMor-PrP than for the *N*-ethyl-substituted compounds. Furthermore, the number of metabolites increased with an increasing length of the alkyl chain in position of the α-carbon (e.g. 4MeO-αP-VP), which was already observed elsewhere (Manier et al. 2018). Both structural properties increase the affinity of metabolizing enzymes to certain cathinones (Manier et al. 2018). An increased α-carbon chain length is also associated with an increased cytotoxicity (Nadal-Gratacos et al. 2023). After *O*-demethylation, sulfation (MO3.9), and glucuronidation (MO3.10) of 4MeO-αP-VP were observed in HepaRG incubations, while *O*-desmethyl glucuronide could not be detected in pHLS9. Other metabolic reactions only detected in HepaRG incubations were hydroxylamine formation (MT1.4) of 4MeS-NE-BP and *N,N*-bis-dealkylation (MT2.5) of 4MeS-αP-BP.

In contrast to a metabolism study of 4MeO-αP-VP using primary human hepatocytes (Ellefsen et al. 2016) or pHLS9 (Richter et al. 2017b), no oxo reduction and its subsequent metabolites could be identified for 4MeO-αP-VP in this study. In comparison to Richter et al., a lactam formation was identified instead and compared to Ellefsen et al., two phase II metabolites. The *N*-dealkyl *O*-desmethyl metabolite postulated in both studies was also identified in pHLS9 and HepaRG, but was of equal abundance as in the negative control of both in vitro systems and was therefore not included. This was also observed for the *N*,*O*-dealkylated metabolite in the negative control of 4MeO-NE-BP; therefore, it was excluded from evaluation. In the negative control of 4MeO-αP-BP and 4MeO-αP-VP, a hydroxy product was identified in both in vitro systems, but the abundance was lower than that in the enzyme incubations. After further evaluation, metabolic formation of both products was therefore assumed.

Comparing HepaRG and pHLS9, the results are in line with literature that both in vitro systems show a similar performance with slightly more metabolites formed in HepaRG (Richter et al. 2017a). As no quantitative data was generated, cells were incubated in a commonly used concentration and no cell viability tests were performed. Nevertheless, potential cytotoxic effects could have reduced the abundance of some low-turnover metabolites. Both positive control incubations (MDMA and quetiapine) showed characteristic phase I and II metabolites in pHLS9 and HepaRG incubations.

### Mass spectra of in vitro metabolites formed by pHLS9 and HepaRG cells

**Fig. 3:**
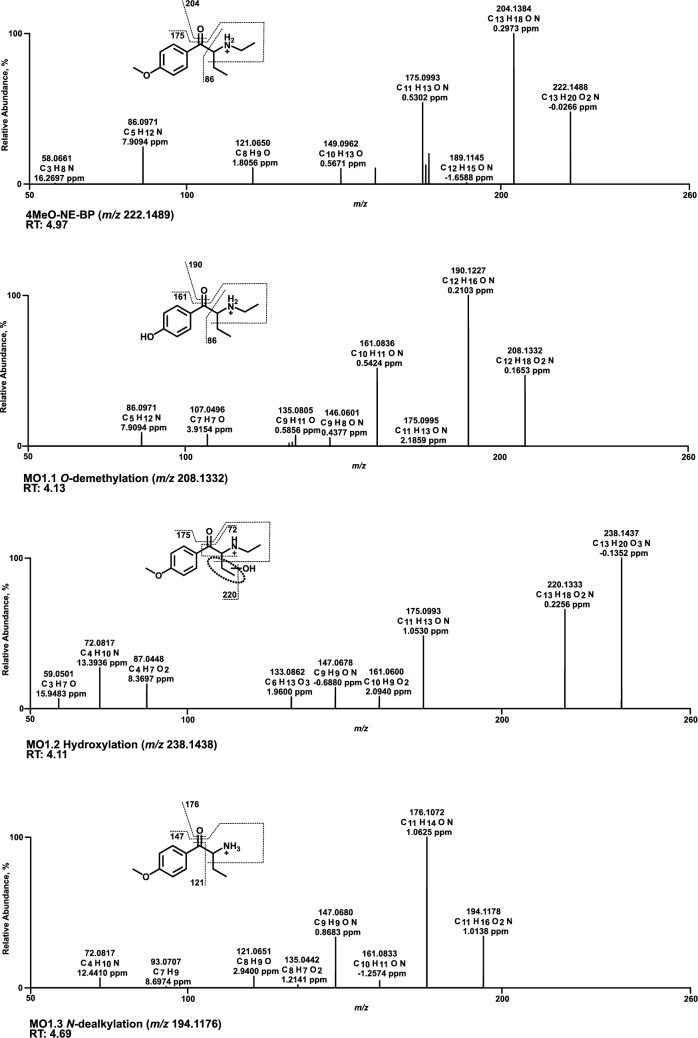
MS^2^ spectra of 4MeO-NE-BP and selected metabolites, sorted by descending abundance

**Fig. 4:**
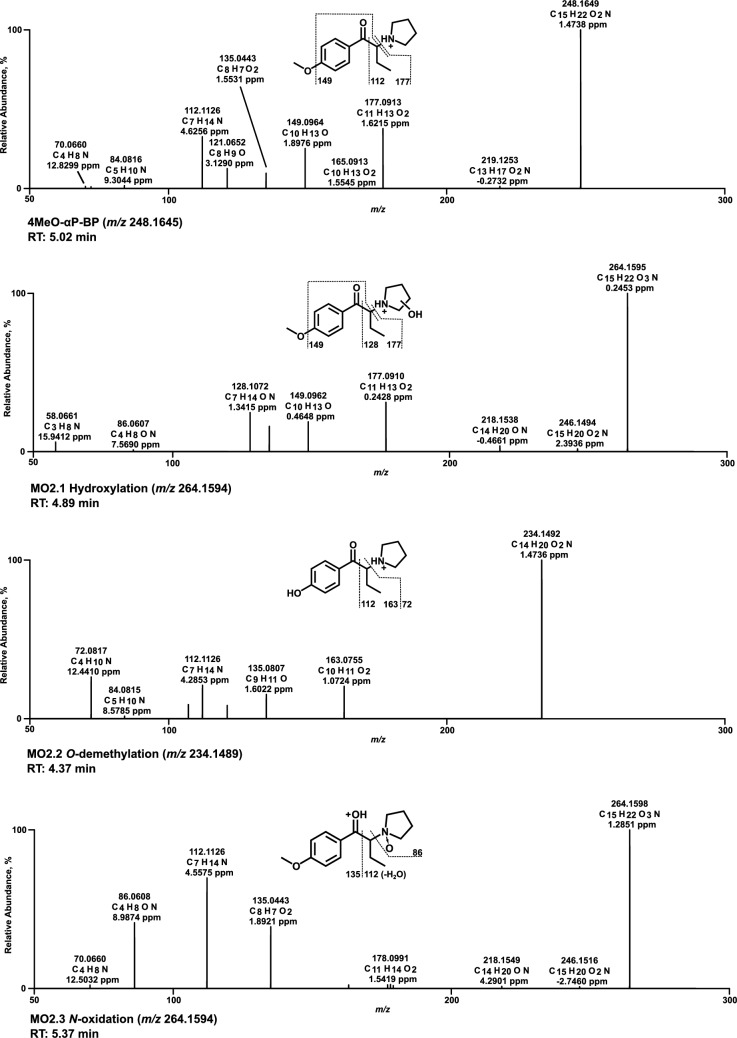
MS^2^ spectra of 4MeO-αP-BP and selected metabolites, sorted by descending abundance

**Fig. 5:**
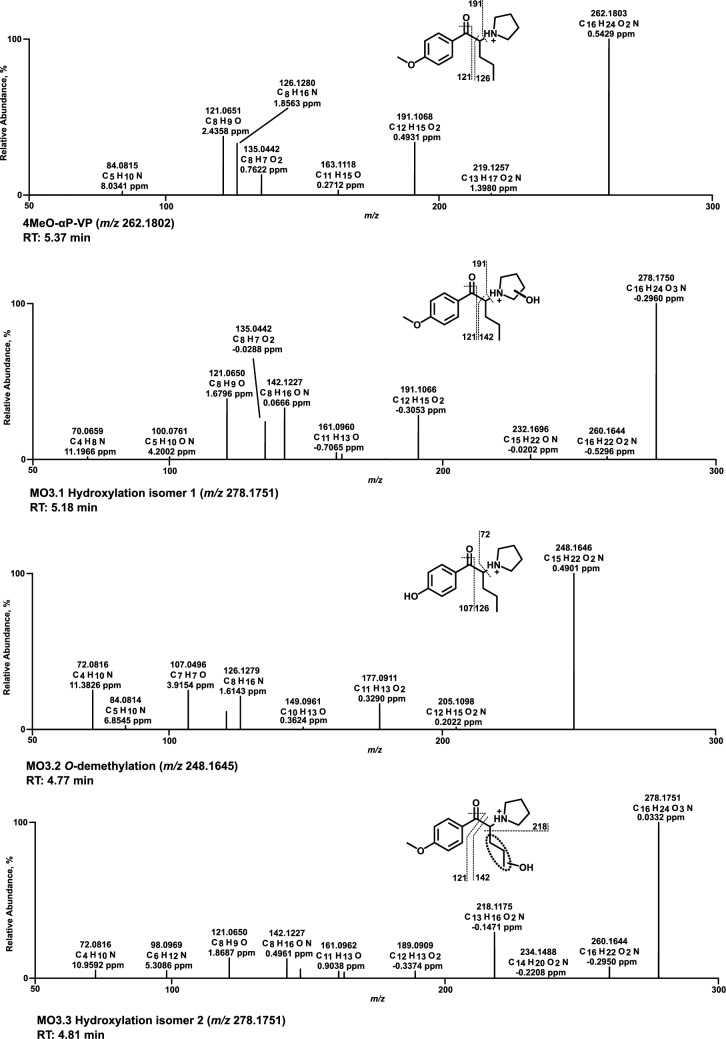
MS^2^ spectra of 4MeO-αP-VP and selected metabolites, sorted by descending abundance

**Fig. 6:**
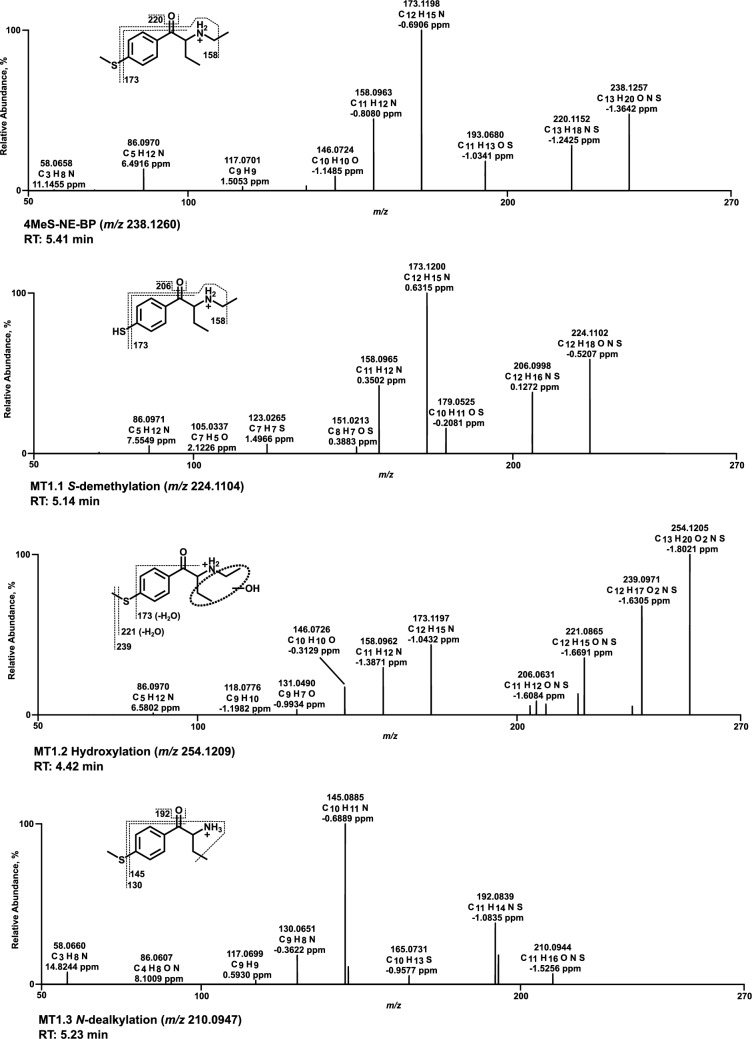
MS^2^ spectra of 4MeS-NE-BP and selected metabolites, sorted by descending abundance

**Fig. 7:**
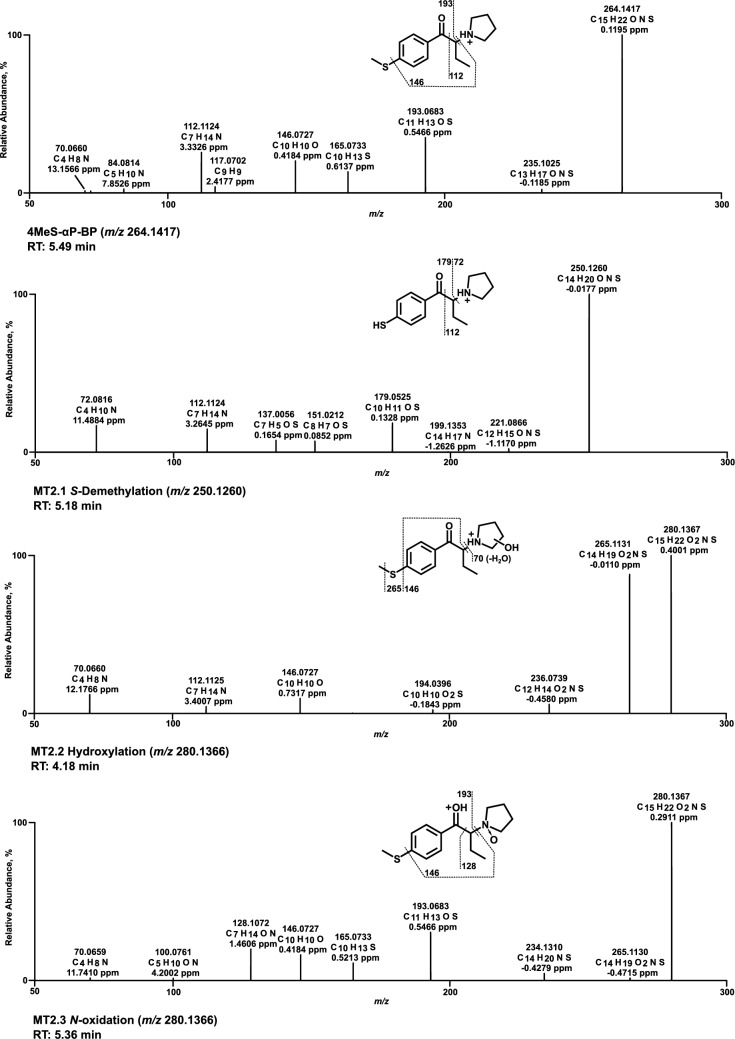
MS^2^ spectra of 4MeS-αP-BP and selected metabolites, sorted by descending abundance

**Fig. 8:**
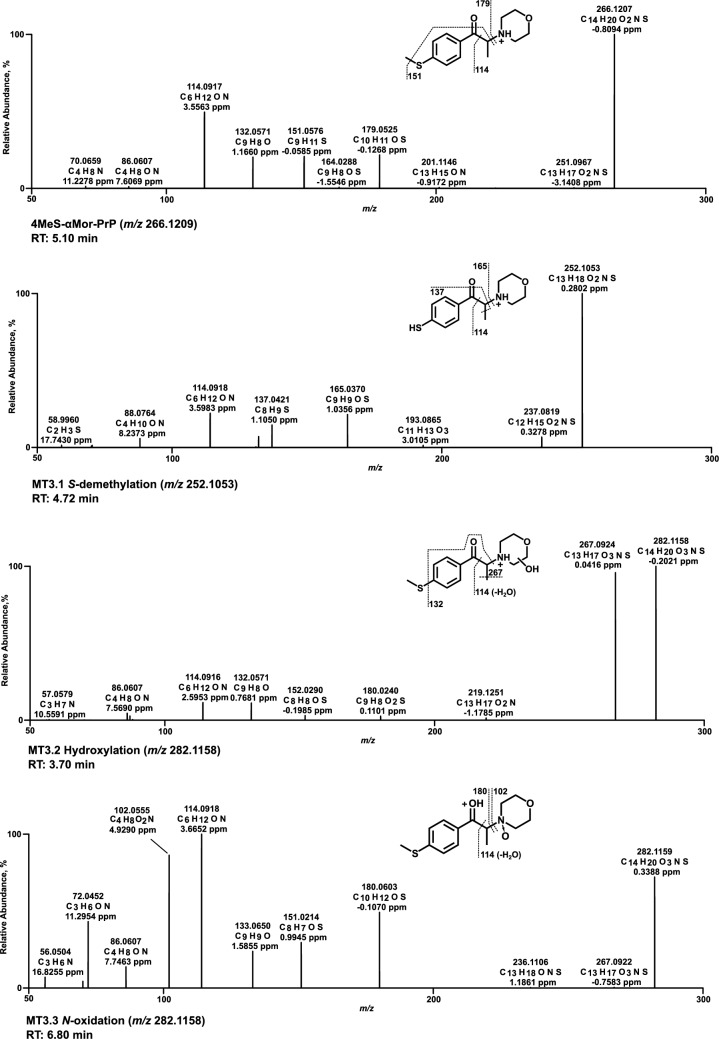
MS^2^ spectra of 4MeS-αMor-PrP and selected metabolites, sorted by descending abundance

All HRMS^2^ spectra are presented using the exact masses of the parent ions (PI) and fragment ions (FI) and all metabolites are considered tentative, as there were no reference standards available. The HRMS^2^ spectrum of each parent compound and its most abundant metabolite were exemplarily described using the three FI with the highest intensity. Metabolic reactions, calculated (exact) *m/z* of the PI and FI, elemental composition, and retention time (RT) are listed in Table S2.1–S2.6 of SI. Spectra are shown in Figs. 3, 4, 5, 6, 7 and 8 and Figs. S6–S11 of SI.

### 4MeO-NE-BP

The spectrum of 4MeO-NE-BP (Fig. 3) showed a PI at *m/z* 222.1489 (C_13_H_20_NO_2_), followed by a cleavage of the carbonyl group oxygen (water loss, FI and base peak at *m/z* 204.1383). Homolytic cleavage of the ethyl group adjacent to the α-carbon resulted in FI at *m/z* 175.0992. Alpha cleavage next to the α-carbon and carbonyl group led to FI at *m/z* 86.0964. Compared to the parent compound, PI of *O*-desmethyl metabolite (MO1.1) at *m/z* 208.1332 was shifted by the mass of a methyl group (−14.0156 u). Fragmentation followed the same pattern as for the parent compound, observing both FI at *m/z* 190.1226 (water loss) and at *m/z* 161.0835 (water loss and homolytic cleavage of the ethyl group), shifted by the mass of a methyl group loss. FI at *m/z* 86.0964 was identical to the parent compound due to alpha cleavage. The hydroxylation of MO1.2 most likely occurred at the ethyl group of the alpha carbon. A key fragment at *m/z* 87.0441 was formed by cleavage at the aromatic system and heterolytic cleavage at the amine. The exact position of hydroxylation within the ethyl group could not be determined.

### 4MeO-αP-BP

Within the spectrum of 4MeO-αP-BP (Fig. 4) with a PI and base peak at *m/z* 248.1645 (C_15_H_22_NO_2_), FI at *m/z* 177.0910 was observed after heterolytic cleavage of the pyrrolidine ring. Followed by pyrrolidine loss, a loss of the methoxy group was detected at *m/z* 149.0961. Alpha cleavage led to FI at *m/z* 112.1121. A similar fragmentation pattern was observed for the hydroxy metabolite (MO2.1) at *m/z* 264.1594. Both FIs of the parent compound at *m/z* 177.0910 and 149.0961 could be detected, and FI at *m/z* 128.1070, which corresponded to FI at *m/z* 112.1121 of the parent spectrum, shifted due to the mass of the hydroxy group. The position of hydroxylation at the pyrrolidine ring of MO2.1 and MO2.5 (Figure S7, SI) could not be concluded from the fragment ions, although the ortho position is favored due to electronic effects. As MS/MS data did not allow differentiation, the hydroxy metabolites were reported without specification of the exact position. In contrast, oxidation is expected to result almost exclusively in the lactam metabolite (MO2.4, Figure S7 of SI). This depiction of the functional groups is consistent with literature data (Manier et al. 2018), (Kavanagh et al. 2020).

### 4MeO-αP-VP

The spectrum of 4MeO-αP-VP (Fig. 5) showed PI and base peak at *m/z* 262.1802 (C_16_H_24_NO_2_). Similar to 4MeO-αP-BP, a heterolytic cleavage of the pyrrolidine ring was observed, resulting in FI at *m/z* 191.1067. Alpha cleavage led to FI at *m/z* 126.1280. FI at *m/z* 121.0648 was observed after alpha cleavage and cleavage of the carbonyl group oxygen (water loss). PI and base peak within the hydroxy metabolite (MO3.1) spectrum were at *m/z* 278.1751. After a heterolytic cleavage of the pyrrolidine ring, FI equal to the parent compound at *m/z* 191.1067 was formed. FI at *m/z* 142.1226 formed due to an alpha cleavage of the parent compound between the α-carbon and the carbonyl group. FI at *m/z* 121.0648 was formed as described for the parent compound. As described for 4MeO-αP-BP, MS/MS data did not allow evaluation of the hydroxylation site. Therefore, the hydroxy metabolite was reported without specification of the exact position at the pyrrolidine ring (MO3.1, Fig. 5). In contrast, oxidation is expected to result almost exclusively in the lactam metabolite (MO3.5, Figure S8 of SI).

### 4MeS-NE-BP

In the spectrum of 4MeS-NE-BP (Fig. 6), PI was detected at *m/z* 238.1260 (C_13_H_20_NOS). After a loss of the oxygen atom of the carbonyl group (water loss, FI at *m/z* 220.1154), the base peak at *m/z* 173.1199 was formed by additional homolytic cleavage of the methylthio group. Alpha cleavage next to the secondary carbon of the *N*-ethyl substituent at *m/z* 173.1199 led to FI at *m/z* 158.0964 (loss of a methyl group). The *S*-desmethyl metabolite (MT1.1) showed the same fragmentation pattern as the parent compound, shifted by the mass of a CH_2_ group (−14.0156 u). The position of hydroxylation of MT1.2 (Fig. 6) and MT 1.7 (Figure S9, SI) could not be determined, as the fragment ions did not provide a clear assignment. Although lactam formation of MT1.5 (Figure S9, SI) appeared most likely, hydroxylation of both ethyl groups appeared equally probable considering electronic and steric aspects. Therefore, no specification of the oxo position was made.

### 4MeS-αP-BP

Within the spectrum of 4MeS-αP-BP (Fig. 7), PI at *m/z* 264.1417 (C_15_H_22_NOS) was observed as the base peak. Heterolytic cleavage of the pyrrolidine ring formed a methylthiophenone FI at *m/z* 193.0682. The counterpart FI to this reaction was the pyrrolidine ring at *m/z* 70.0651. After the pyrrolidine loss, a homolytic cleavage of the methylthio group resulted in FI at *m/z* 146.0726. The propylpyrrolidine FI at *m/z* 112.1121 was formed by alpha cleavage of the parent compound between the carbonyl group and the α-carbon. The *S*-desmethyl metabolite (MT2.1) PI was at *m/z* 250.1260. Heterolytic cleavage of the pyrrolidine ring resulted in both FI at *m/z* 179.0525 for the phenone derivative and FI of the pyrrolidine ring at *m/z* 72.0808. FI at *m/z* 112.1121 was formed as described above for the parent compound. The hydroxy metabolites MT2.2 (Fig. 7) and MT2.6 (Figure S10, SI) were reported without specification of the exact position at the pyrrolidine ring, as MS/MS data did not allow an assignment. In contrast, oxidation is expected to result almost exclusively in the lactam metabolite (MT2.4, Figure S10 of SI).

### 4MeS-αMor-PrP

In the spectrum of 4MeS-αMor-PrP (Fig. 8), the PI and base peak were at *m/z* 266.1209 (C_14_H_20_NO_2_S). A morpholine ring loss resulted in the methylthiophenone FI at *m/z* 179.0525. FI at *m/z* 151.0576 was derived from FI at *m/z* 179.0525 after a loss of the carbonyl group oxygen (water loss) and a homolytic cleavage of the methyl group within the methylthio group. Alpha cleavage led to the ethyl morpholine FI at *m/z* 114.0913. The base peak and PI in the spectrum of the *S*-desmethyl metabolite (MT3.1) were detected at *m/z* 252.1053. The morpholine ring loss resulted in FI at *m/z* 165.0369, shifted by a mass of −14.0156 u (methyl group loss) compared to the parent spectrum. Following morpholine ring loss, the loss of the carbonyl group oxygen and the homolytic cleavage of the methyl group next to the α-carbon resulted in FI at *m/z* 137.0419. FI at *m/z* 114.0913 was formed as described above for the parent compound. The hydroxy metabolites MT3.2 (Fig. 8) and MT3.5 (Figure S11, SI) were reported without specification of the exact position at the morpholine ring, as MS/MS data did not allow an assignment. In contrast, oxidation is expected to result almost exclusively in the lactam metabolite (MT3.6, Figure S11 of SI).

### Monooxygenases activity screening

In a monooxygenases activity screening, metabolites were tentatively identified in separate incubations. Additionally, isozymes involved in the formation of a one-step metabolite can be identified. Both allows an assessment of potential drug–drug interactions. Such cases are known for NPS, for example, a mechanism-based inhibition of CYP2D6 by methylenedioxy stimulants (de la Torre et al. 2012). If the metabolic pathways are known, increased risk of intoxication due to polymorphisms can be assessed. The initial metabolic reactions, metabolite-IDs, and detectability of the investigated monooxygenases are shown in Tables S3.1 − S3.6 of SI.

Monooxygenases activity screening revealed extensive metabolism by numerous monooxygenases and, thus, increased toxicity due to drug–drug interactions or polymorphisms is not expected. However, the extent to which active metabolites could potentially contribute to toxic effects is currently unknown. Examples involving other synthetic cathinones have been described previously (Mayer et al. 2016; Elmore et al. 2017). All cathinones were mainly metabolized by CYP2C9, CYP2C19, CYP2D6, and CYP3A4, which is consistent with other literature reports (Manier et al. 2018). *N*-Oxidation or hydroxylamine formation of every compound was catalyzed by FMO3, among other monooxygenases. Some metabolic pathways were only mediated by one CYP isoform, such as the *S*-demethylation of 4MeS-NE-BP and 4MeS-αP-BP via CYP2D6. No differences could be seen in the isoforms involved in the metabolic reactions, neither within nor between the two compound groups.

## Conclusions

In this study, the in vitro toxicokinetics of three 4’-methoxy and three 4’-methylthio-substituted cathinones were investigated including plasma protein binding, metabolism via pHLS9 and HepaRG, and monooxygenases activity screening. Plasma protein binding of 4MeO-NE-BP and 4MeO-αP-BP was low and moderate for the methylthio compounds and 4MeO-αP-VP, which could potentially lead to an increased in vivo half-life of the methylthio compounds. The parent compound and the hydroxy, *S*- or *O*-desmethyl metabolites were the most abundant for all compounds and are therefore recommended as toxicological routine screening targets, especially if sample preparation includes conjugate cleavage. This should yet be confirmed in human samples. An increasing length of the α-carbon alkyl chain and a heterocyclic instead of alkyl substitution increased the number of tentatively identified metabolites. Both in vitro models yielded a similar number of total metabolites. Thus, pHLS9 can be recommended for initial screening due to its cost-effectiveness. However, in addition to the slightly improved performance, the use of HepaRG may be regarded as a superior in vitro metabolic system due to its increased complexity, which allows for the consideration of transmembrane transport processes. Based on the results of the monooxygenases activity screening, drug–drug interactions or increased toxicity due to polymorphisms are unlikely. Further studies should include tests on cell viability and cytochrome P450 inhibition to provide more details of toxicity at a cellular level. In vivo studies using microdosing approaches would be valuable to confirm suggested biomarkers.

## Supplementary Information

Below is the link to the electronic supplementary material.

Supplementary file1 (PDF 6159 KB)

## Data Availability

Data might be made available upon reasonable request from the corresponding author.
