# Supplementary material for: In vitro toxicokinetics and metabolic profiling of methoxycathinones and methylthiocathinones using human liver systems and hyphenated mass spectrometry
Source: Arch Toxicol. 2025 Sep 29;100(1):275–89. doi: 10.1007/s00204-025-04205-x (PMC12858467; doi:10.1007/s00204-025-04205-x)
Supplement: Supplementary file 1 — Supplementary file1 (PDF 6159 KB) [file 204_2025_4205_MOESM1_ESM.pdf]

Supplementary Information

In Vitro Toxicokinetics and Metabolic Profiling of Methoxycathinones and Methylthiocathinones Using Human Liver Systems and Hyphenated Mass Spectrometry

Matthias D. Kroesen[1], *Tanja M. Gampfer*[1], *Lea Wagmann*[1], *Pierce V. Kavanagh*[2], *Simon D. Brandt*[3], *Markus R. Meyer**

[1]*Department of Experimental and Clinical Toxicology and Pharmacology, Center for Molecular Signaling (PZMS), PharmaScienceHub (PSH), Saarland University, Homburg, Germany*

[2]*Department of Pharmacology and Therapeutics, School of Medicine, Trinity Centre for Health Sciences, St. James Hospital, Dublin 8, Ireland*

[3]*The Alexander Shulgin Research Institute, 1483 Shulgin Road, Lafayette, CA 94549, USA*

**Correspondence:* m.r.meyer@mx.uni-saarland.de

HepaRG incubation conditions

HepaRG cells were thawed and resuspended in 13.5 mL (~ 740,000 cells/mL) thaw and seed medium at 37 °C. Thaw and seed medium consisted of Williams E medium, 100 U/mL penicillin, 100 µg/mL streptomycin, GlutaMAX, and HPRG670 supplement. Cells were handled under sterile conditions using a laminar flow bench class II (Thermo Scientific, TF, Schwerte, Germany). Aliquots of 100 µL cell suspension were seeded on collagen-coated 96-well plates (~ 74,000 cells/well). Evaporation was minimized by filling the outer wells with 100 µL thaw and seed medium. Cells were preincubated for 4 h in an incubator (Binder, Tuttlingen, Germany) at 37 °C, 95 % air humidity, and 5 % CO_2. After preincubation, 50 µL of the thaw and seed medium was replaced with 50 µL solution of each cathinone (25 µM) in thaw and seed medium, followed by an incubation for 24 h at 37 °C, 95 % air humidity, and 5 % CO_2 atmosphere.

LC-HRMS/MS apparatus and conditions

A Thermo Fisher (TF, Dreieich, Germany) Dionex UltiMate 3000 RS pump consisting of a degasser, a quaternary pump, and an autosampler, coupled to a TF Q Exactive Plus system equipped with a heated electrospray ionization (HESI)-II source were used. A mass calibration was done according to the manufacturer's recommendations using external mass calibration prior to analysis. Gradient elution was performed on a TF Accucore PhenylHexyl column (100 mm x 2.1 mm, 2.6 µm) with a 2 mM aqueous ammonium formate solution containing 0.1 % (*v/v*) formic acid (pH 3, eluent A) and 2 mM ammonium formate solution in acetonitrile/methanol (50:50, *v/v*) containing 0.1 % (*v/v*) formic acid, and 1 % (*v/v*) water (eluent B). The gradient was stepped as follows: 0–2.5 min hold 99 % A, 2.5–8 min to 1 % A, 8–9.5 min hold 1 % A, and 9.5–11.5 min hold 99 % A. Initial flow rate from 0–9.5 min was 500 µL/min and final flow rate was 800 µL/min from 9.5–11.5 min. Mass spectrometry was performed using full scan data and a subsequent data-dependent acquisition (DDA) with priority to mass-to-charge ratios (*m/z*) of parent compounds and their expected metabolites. The inclusion list contained *m/z* values of likely formed metabolites such as *O*- or *S*-desmethyl, hydroxy, and oxo reduction metabolites (phase I) as well as sulfates, glucuronides (phase II), and combinations thereof. Chemdraw 23.1.1 was used to draw structures of expected metabolites and for exact mass calculations. TF Xcalibur Qual Browser software version 4.6 was used for data handling. Mass deviations of the parent compound were

accepted up to 5 ppm. Plasma protein binding samples were measured only in positive ionization mode, all other samples were measured in positive and negative ionization mode. Instrument settings, data generation settings, and data evaluation settings are shown in **Table S1**.

Table S1. Instrument settings for analysis using the TF Q Exactive Plus system.

**HESI-II source conditions**	
Heater temperature	320 °C
Ion transfer capillary temperature	320 °C
Spray voltage	4.0 kV
Ionization mode	Positive and negative
Sheath gas	60 arbitrary units (AU)
Auxiliary gas	10 AU
Sweep gas	0 AU
S-lens RF level	50.0
**Full scan data acquisition**	
Resolution	35,000
Microscans	1
Automatic gain control (AGC) target	1e6
Maximum injection time (IT)	120 ms
Scan range	*m/z* 50-750
**Settings for DDA mode plus inclusion list**	
Option “pick others”	Enabled
Dynamic exclusion	0.1 s
Resolution	17,500
Microscans	1
Isolation window	1.0 *m/z*
Loop count	5
AGC target	2e5
Maximum IT	250 ms
High collision dissociation cell with stepped normalized collision energy	17.5, 35.0, 52.5
Exclude isotopes	On
Spectrum data type	Profile
Underfill ratio	1 %
**Peak integration**	
Peak detection algorithm	INCOS
Baseline window	40
Area noise factor	5
Peak noise factor	10

Table S2.1 Detection of 4MeO-NE-BP (4′-methoxy-*N*-ethylbutyrophenone) and its phase I and phase II metabolic reactions in pooled human liver S9 fraction, HepaRG and/or isozyme incubations together with their metabolite identification numbers (ID), the calculated exact mass of the protonated parent ion ($M + H^+$) and its three most abundant fragment ions (FI 1-3), elemental composition, retention time (RT) recorded in $HRMS^2$ mode. Metabolites were sorted by descending abundance.

Metabolite-ID	Metabolic reaction	Calculated exact mass, *m/z*	Elemental composition	RT, min	FI 1, *m/z*	FI 2, *m/z*	FI 3, *m/z*
4MeO-NE-BP		222.1489	$C_{13}H_{20}NO_2$	4.97	204.1383	175.0992	86.0964
MO1.1	*O*-Demethylation	208.1332	$C_{12}H_{18}NO_2$	4.13	190.1226	161.0835	86.0964
MO1.2	Hydroxylation	238.1438	$C_{13}H_{20}NO_3$	4.11	220.1332	175.0992	72.0808
MO1.3	*N*-Dealkylation	194.1176	$C_{11}H_{16}NO_2$	4.69	176.1070	147.0679	121.0684
MO1.4	Hydroxylamine formation	238.1438	$C_{13}H_{20}NO_3$	5.50	135.0441	102.0913	84.0808
MO1.5	*O*-Demethylation + sulfation	288.0900	$C_{12}H_{18}NO_5S$	3.91	190.1226	208.1332	161.0835

Table S2.2 Detection of 4MeO-αP-BP (4′-methoxy-α-pyrrolidinobutyrophenone) and its phase I metabolic reactions in pooled human liver S9 fraction, HepaRG and/or isozyme incubations together with their metabolite identification numbers (ID), the calculated exact mass of the protonated parent ion ($M + H^+$) and its three most abundant fragment ions (FI 1-3), elemental composition, retention time (RT) recorded in $HRMS^2$ mode. Metabolites were sorted by descending abundance.

Metabolite-ID	Metabolic reaction	Calculated exact mass, *m/z*	Elemental composition	RT, min	FI 1, *m/z*	FI 2, *m/z*	FI 3, *m/z*
4MeO-αP-BP		248.1645	$C_{15}H_{22}NO_2$	5.02	177.0910	112.1121	149.0961
MO2.1	Hydroxylation	264.1594	$C_{15}H_{22}NO_3$	4.89	177.0910	128.1070	149.0961
MO2.2	*O*-Demethylation	234.1489	$C_{14}H_{20}NO_2$	4.37	163.0754	72.0808	112.1121
MO2.3	*N*-Oxidation	264.1594	$C_{15}H_{22}NO_3$	5.37	112.1121	86.0600	135.0441
MO2.4	Lactam formation	262.1438	$C_{15}H_{20}NO_3$	6.44	126.0913	154.0863	177.0910
MO2.5	Hydroxylation + *N*-oxidation	280.1543	$C_{15}H_{22}NO_4$	4.98	126.0913	262.1438	176.1070

Table S2.3 Detection of 4MeO-αP-VP (4′-methoxy-α-pyrrolidinovalerophenone) and its phase I and phase II metabolic reactions in pooled human liver S9 fraction, HepaRG and/or isozyme incubations together with their metabolite identification numbers (ID), the calculated exact mass of the protonated parent ion ($M + H^+$) and its three most abundant fragment ions (FI 1-3), elemental composition, retention time (RT) recorded in $HRMS^2$ mode. Metabolites were sorted by descending abundance.

Metabolite-ID	Metabolic reaction	Calculated exact mass, *m/z*	Elemental composition	RT, min	FI 1, *m/z*	FI 2, *m/z*	FI 3, *m/z*
4MeO-αP-VP		262.1802	$C_{16}H_{24}NO_2$	5.37	121.0648	191.1067	126.1280
MO3.1	Hydroxylation isomer 1	278.1751	$C_{16}H_{24}NO_3$	5.18	121.0648	142.1226	191.1067
MO3.2	*O*-Demethylation	248.1645	$C_{15}H_{22}NO_2$	4.77	107.0491	72.0808	126.1280
MO3.3	Hydroxylation isomer 2	278.1751	$C_{16}H_{24}NO_3$	4.81	218.1176	121.0648	142.1226
MO3.4	*N*-Oxidation	278.1751	$C_{16}H_{24}NO_3$	5.64	126.1280	86.0600	135.0441
MO3.5	Lactam formation	276.1594	$C_{16}H_{22}NO_3$	6.68	140.1070	98.0600	121.0648
MO3.6	*O*-Demethylation + hydroxylation	264.1594	$C_{15}H_{22}NO_3$	4.47	72.0808	126.1280	193.0859
MO3.7	Hydroxylation + *N*-oxidation	294.1700	$C_{16}H_{24}NO_4$	5.24	276.1594	140.1070	121.0648
MO3.8	*O*-Demethylation + dihydroxylation	280.1543	$C_{15}H_{22}NO_4$	4.66	262.1438	176.1070	87.0441
MO3.9	*O*-Demethylation + sulfation	328.1213	$C_{15}H_{22}NO_5S$	4.68	248.1645	107.0491	177.0910
MO3.10	*O*-Demethylation + glucuronidation	424.1966	$C_{21}H_{30}NO_8$	3.91	248.1645	177.0910	107.0491

Table S2.4 Detection of 4MeS-NE-BP (4′-methylthio-*N*-ethylbutyrophenone) and its phase I metabolic reactions in pooled human liver S9 fraction, HepaRG and/or isozyme incubations together with their metabolite identification numbers (ID), the calculated exact mass of the protonated parent ion ($M + H^+$) and its three most abundant fragment ions (FI 1-3), elemental composition, retention time (RT) recorded in $HRMS^2$ mode. Metabolites were sorted by descending abundance.

Metabolite-ID	Metabolic reaction	Calculated exact mass, *m/z*	Elemental composition	RT, min	FI 1, *m/z*	FI 2, *m/z*	FI 3, *m/z*
4MeS-NE-BP		238.1260	$C_{13}H_{20}NOS$	5.41	173.1199	158.0964	220.1154
MT1.1	*S*-Demethylation	224.1104	$C_{12}H_{18}NOS$	5.14	173.1199	158.0964	206.0998
MT1.2	Hydroxylation	254.1209	$C_{13}H_{20}NO_2S$	4.42	239.0975	173.1199	221.0869
MT1.3	*N*-Dealkylation	210.0947	$C_{11}H_{16}NOS$	5.23	145.0886	192.0839	130.0651
MT1.4	Hydroxylamine formation	254.1209	$C_{13}H_{20}NO_2S$	6.23	151.0212	102.0913	84.0808
MT1.5	Hydroxylation + oxidation	252.1053	$C_{13}H_{18}NO_2S$	6.46	151.0212	84.0808	136.0341
MT1.6	*N*-Dealkylation + *S*-Demethylation	196.0791	$C_{10}H_{14}NOS$	4.88	145.0886	178.0685	179.0525
MT1.7	Hydroxylamine formation + hydroxylation	270.1158	$C_{13}H_{20}NO_3S$	5.50	158.0963	173.1198	146.0726

Table S2.5 Detection of 4MeS-αP-BP (4′-methylthio-α-pyrrolidinobutyrophenone) and its phase I metabolic reactions in pooled human liver S9 fraction, HepaRG and/or isozyme incubations together with their metabolite identification numbers (ID), the calculated exact mass of the protonated parent ion ($M + H^+$) and its three most abundant fragment ions (FI 1-3), elemental composition, retention time (RT) recorded in $HRMS^2$ mode. Metabolites were sorted by descending abundance.

**Metabolite-ID**	**Metabolic reaction**	**Calculated exact mass, *m/z***	**Elemental composition**	**RT, min**	**FI 1, *m/z***	**FI 2, *m/z***	**FI 3, *m/z***
4MeS-αP-BP		264.1417	$C_{15}H_{22}NOS$	5.49	193.0682	112.1121	146.0726
MT2.1	*S*-Demethylation	250.1260	$C_{14}H_{20}NOS$	5.18	179.0525	72.0808	112.1121
MT2.2	Hydroxylation	280.1366	$C_{15}H_{22}NO_2S$	4.18	265.1131	70.0651	146.0726
MT2.3	*N*-Oxidation	280.1366	$C_{15}H_{22}NO_2S$	5.36	193.0682	128.1070	146.0726
MT2.4	Lactam formation	278.1209	$C_{15}H_{20}NO_2S$	6.77	126.0913	193.0682	146.0726
MT2.5	*N*,*N*-bis-dealkylation	210.0947	$C_{11}H_{16}NOS$	5.22	145.0886	192.0841	130.0651
MT2.6	Dihydroxylation	296.1315	$C_{15}H_{22}NO_3S$	4.35	146.0727	112.1121	70.0651
MT2.7	*S*-Demethylation + hydroxylation	266.1209	$C_{14}H_{20}NO_2S$	5.06	146.0726	112.1121	72.0808
MT2.8	*N*-Oxidation + hydroxylation	296.1315	$C_{15}H_{22}NO_3S$	5.32	231.1254	126.0913	216.1019

Table S2.6 Detection of 4MeS-αMor-PrP (4′-methylthio-2-morpholinopropiophenone) and its phase I metabolic reactions in pooled human liver S9 fraction, HepaRG and/or isozyme incubations together with their metabolite identification numbers (ID), the calculated exact mass of the protonated parent ion (M + H$^+$) and its three most abundant fragment ions (FI 1-3), elemental composition, retention time (RT) recorded in HRMS^2 mode. Metabolites were sorted by descending abundance.

**Metabolite-ID**	**Metabolic reaction**	**Calculated exact mass, *m/z***	**Elemental composition**	**RT, min**	**FI 1, *m/z***	**FI 2, *m/z***	**FI 3, *m/z***
4MeS-αMor-PrP		266.1209	$C_{14}H_{20}NO_2S$	5.10	114.0913	179.0525	151.0576
MT3.1	*S*-Demethylation	252.1053	$C_{13}H_{18}NO_2S$	4.72	114.0913	165.0369	137.0419
MT3.2	Hydroxylation	282.1158	$C_{14}H_{20}NO_3S$	3.70	267.0924	114.0913	132.0570
MT3.3	*N*-Oxidation	282.1158	$C_{14}H_{20}NO_3S$	6.80	114.0913	102.0550	180.0603
MT3.4	Oxo reduction	268.1366	$C_{14}H_{22}NO_2S$	5.00	250.1260	235.1025	165.0732
MT3.5	Hydroxylation + *N*-oxidation	298.1108	$C_{14}H_{20}NO_4S$	5.13	144.0808	233.1046	280.1002
MT3.6	Lactam formation	280.1002	$C_{14}H_{18}NO_3S$	6.34	128.0706	100.0757	156.0655
MT3.7	*N,O*-bis-dealkylation	240.1053	$C_{12}H_{18}NO_2S$	4.93	175.0992	144.0808	222.0947
MT3.8	S-Demethylation + oxo reduction	254.1209	$C_{13}H_{20}NO_2S$	4.56	236.1104	221.0869	151.0576
MT3.9	Dehydrogenation	264.1053	$C_{14}H_{18}NO_2S$	5.21	236.1104	85.0528	151.0213
MT3.10	*N,O*-Dealkylation + *S*-demethylation	226.0896	$C_{11}H_{16}NO_2S$	4.40	175.0992	208.0791	144.0808

Table S3.1 Monooxygenases activity screening of the one-step metabolites of 4MeO-NE-BP. Metabolite-IDs are in ascending order and refer to Table S2.1. Cytochrome P450 (CYP); flavin-containing monooxygenase 3 (FMO3); +, detected; -, not detected.

Metabolic reactions (metabolite-ID)	Enzyme incubations										
	1A2	2A6	2B6	2C8	2C9	2C19	2D6	2E1	3A4	3A5	FMO3
O-Demethylation (MO1.1)	+	-	-	-	-	+	+	-	-	-	-
Hydroxylation (MO1.2)	+	-	+	-	-	+	+	-	-	-	-
N-Dealkylation (MO1.3)	-	-	+	-	-	+	-	-	+	-	-
Hydroxylamine formation (MO1.4)	+	-	+	+	-	+	+	-	+	+	+

Table S3.2 Monooxygenases activity screening of the one-step metabolites of 4MeO-αP-BP. Metabolite-IDs are in ascending order and refer to Table S2.2. Cytochrome P450 (CYP); flavin-containing monooxygenase 3 (FMO3); +, detected; -, not detected.

Metabolic reactions (metabolite-ID)	Enzyme incubations										
	1A2	2A6	2B6	2C8	2C9	2C19	2D6	2E1	3A4	3A5	FMO3
Hydroxylation (MO2.1)	+	-	+	+	-	+	-	-	-	-	-
O-Demethylation (MO2.2)	+	-	-	-	-	+	+	-	-	-	-
N-Oxidation (MO2.3)	+	-	-	-	-	-	+	-	+	+	+

Table S3.3 Monooxygenases activity screening of the one-step metabolites of 4MeO-αP-VP. Metabolite-IDs are in ascending order and refer to Table S2.3. Cytochrome P450 (CYP); flavin-containing monooxygenase 3 (FMO3); +, detected; -, not detected.

Metabolic reactions (metabolite-ID)	Enzyme incubations										
	1A2	2A6	2B6	2C8	2C9	2C19	2D6	2E1	3A4	3A5	FMO3
Hydroxylation isomer 1 (MO3.1)	-	-	+	+	+	+	-	-	-	-	-
O-Demethylation (MO3.2)	+	-	-	+	-	+	+	-	+	-	-
Hydroxylation isomer 2 (MO3.3)	+	-	+	+	-	+	-	-	+	+	-
N-Oxidation (MO3.4)	+	+	-	-	-	-	+	-	+	-	+

Table S3.4 Monooxygenases activity screening of the one-step metabolites of 4MeS-NE-BP. Metabolite-IDs are in ascending order and refer to Table S2.4. Cytochrome P450 (CYP); flavin-containing monooxygenase 3 (FMO3); +, detected; -, not detected.

Metabolic reactions (metabolite-ID)	Enzyme incubations										
	1A2	2A6	2B6	2C8	2C9	2C19	2D6	2E1	3A4	3A5	FMO3
S-Demethylation (MT1.1)	-	-	-	-	-	-	+	-	-	-	-
Hydroxylation (MT1.2)	+	-	-	-	-	+	+	-	+	+	-
N-Dealkylation (MT1.3)	+	-	+	+	-	+	+	-	+	+	-
Hydroxylamine formation (MT1.4)	+	-	-	-	-	-	+	-	+	+	+

Table S3.5 Monooxygenases activity screening of the one-step metabolites of 4MeS-αP-BP. Metabolite-IDs are in ascending order and refer to Table S2.5. Cytochrome P450 (CYP); flavin-containing monooxygenase 3 (FMO3); +, detected; -, not detected.

Metabolic reactions (metabolite-ID)	Enzyme incubations										
	1A2	2A6	2B6	2C8	2C9	2C19	2D6	2E1	3A4	3A5	FMO3
S-Demethylation (MT2.1)	-	-	-	-	-	-	+	-	-	-	-
Hydroxylation (MT2.2)	+	-	+	-	-	+	+	-	+	+	-
N-Oxidation (MT2.3)	-	-	-	-	-	-	-	-	-	-	+

Table S3.6 Monooxygenases activity screening of the one-step metabolites of 4MeS-αMor-PrP. Metabolite-IDs are in ascending order and refer to Table S2.6. Cytochrome P450 (CYP); flavin-containing monooxygenase 3 (FMO3); +, detected; -, not detected.

Metabolic reactions (metabolite-ID)	Enzyme incubations										
	1A2	2A6	2B6	2C8	2C9	2C19	2D6	2E1	3A4	3A5	FMO3
S-Demethylation (MT3.1)	+	-	+	+	-	+	+	-	+	+	-
Hydroxylation (MT3.2)	-	+	+	+	+	+	+	-	+	+	-
N-Oxidation (MT3.3)	-	+	-	-	-	-	-	-	-	-	+

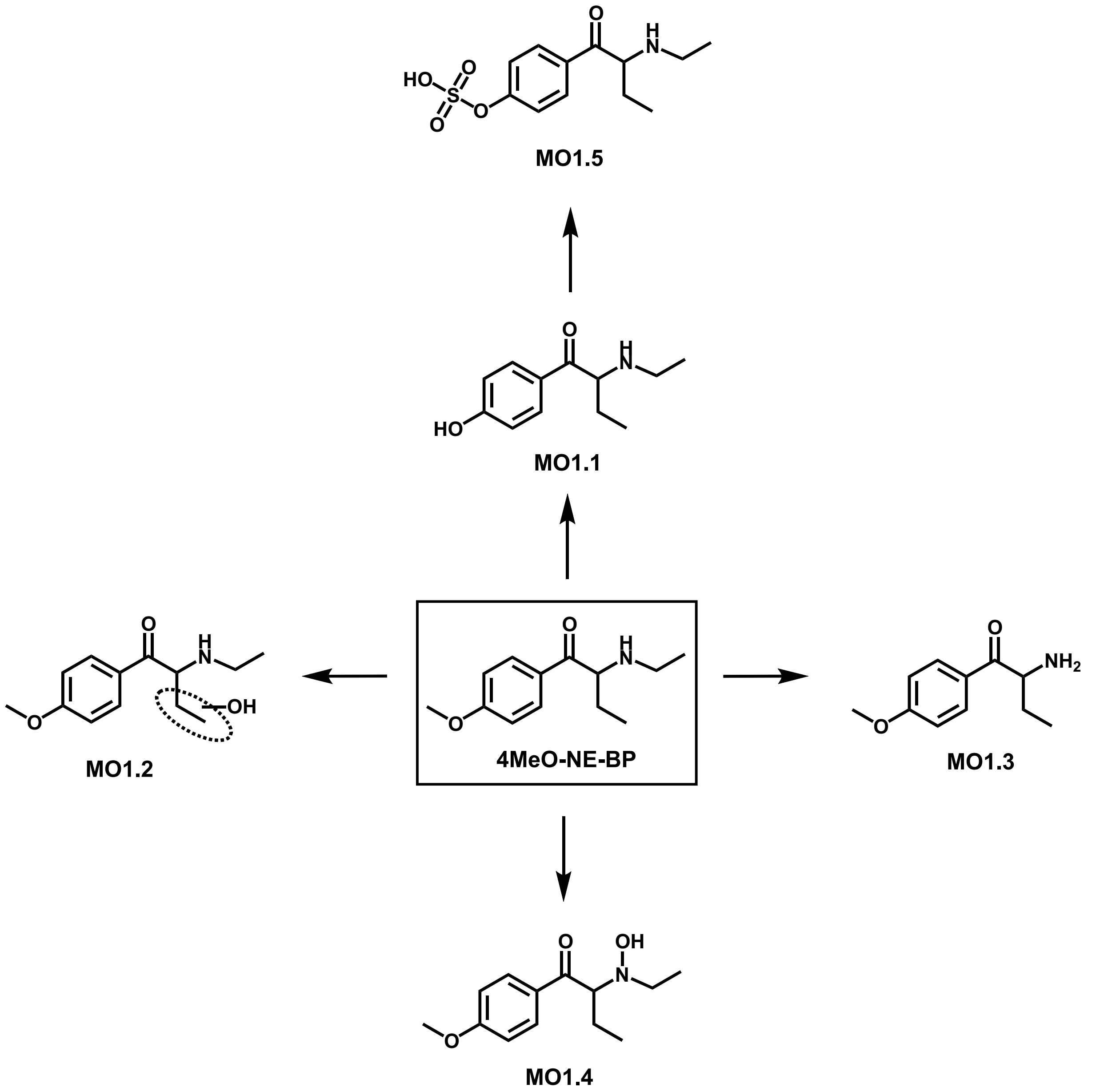

Figure S1 In vitro metabolic pathways of 4MeO-NE-BP in HepaRG, pHLS9 and/or monooxygenases incubations. Metabolite-IDs refer to Table S2.1. →, metabolized to.

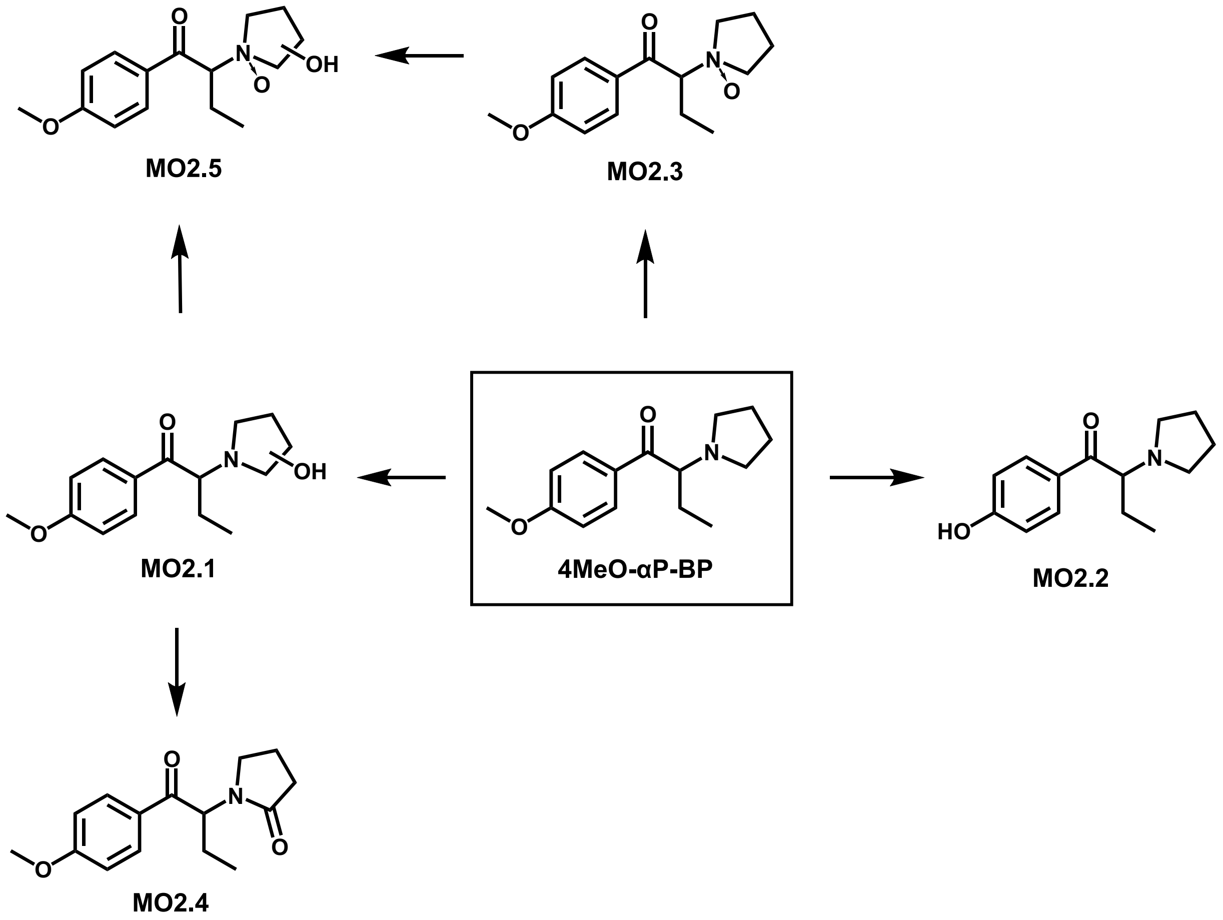

Figure S2 In vitro metabolic pathways of 4MeO-αP-BP in HepaRG, pHLS9 and/or monooxygenases incubations. Metabolite-IDs refer to Table S2.2. →, metabolized to.

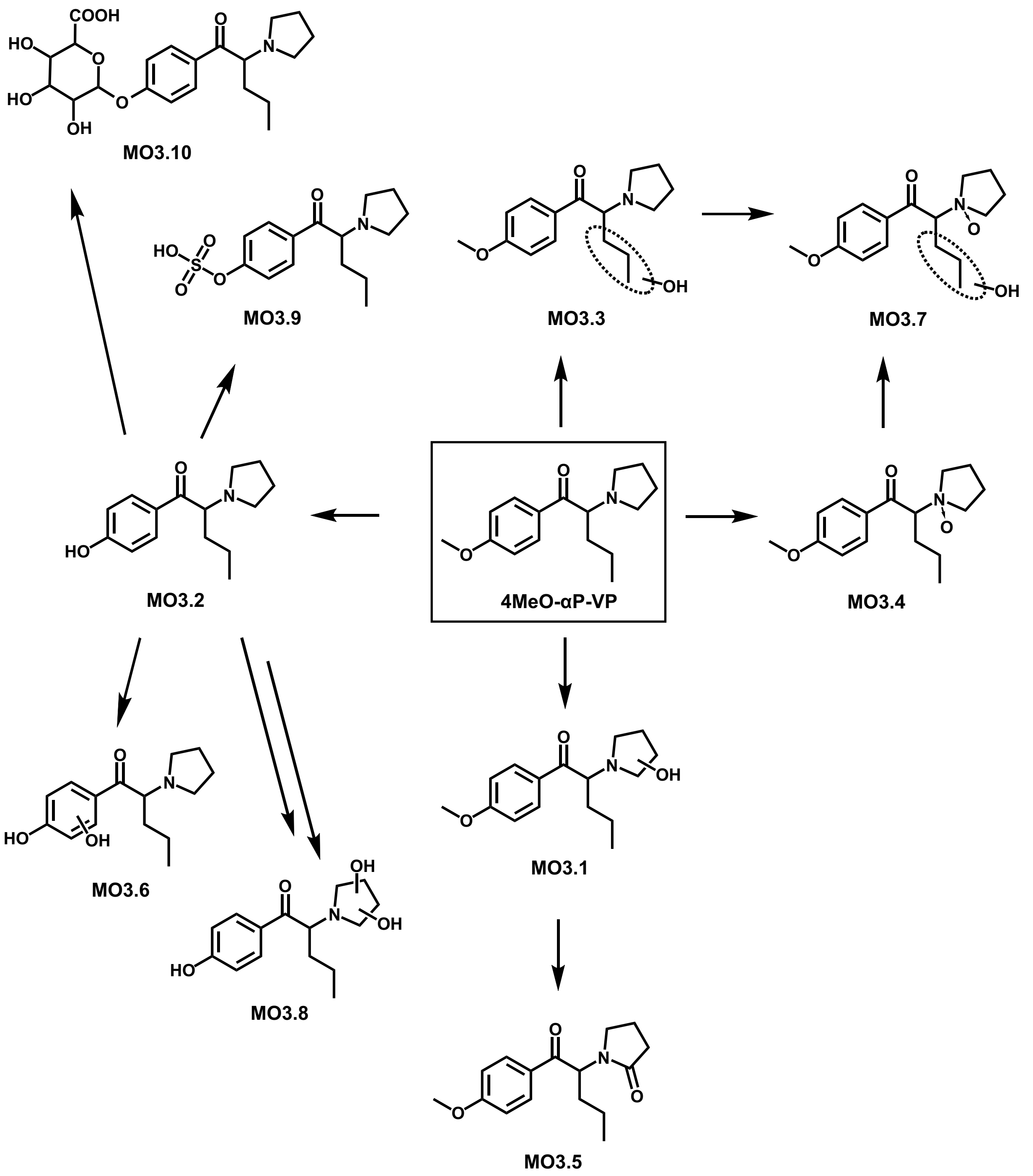

Figure S3 In vitro metabolic pathways of 4MeO-αP-VP in HepaRG, pHLS9 and/or monooxygenases incubations. Metabolite-IDs refer to Table S2.3. →, metabolized to.

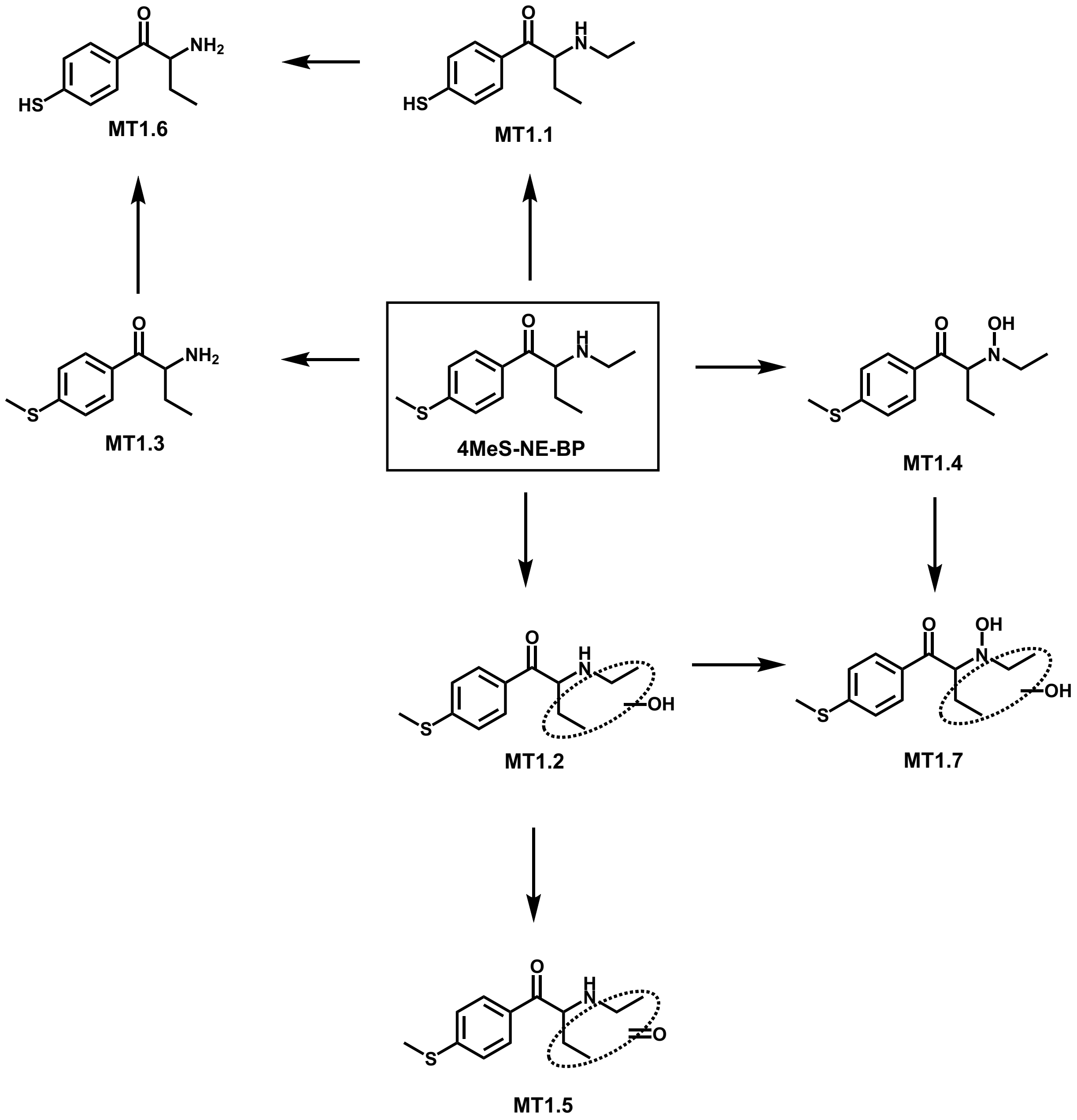

Figure S4 In vitro metabolic pathways of 4MeS-NE-BP in HepaRG, pHLS9 and/or monooxygenases incubations. Metabolite-IDs refer to Table S2.4. →, metabolized to.

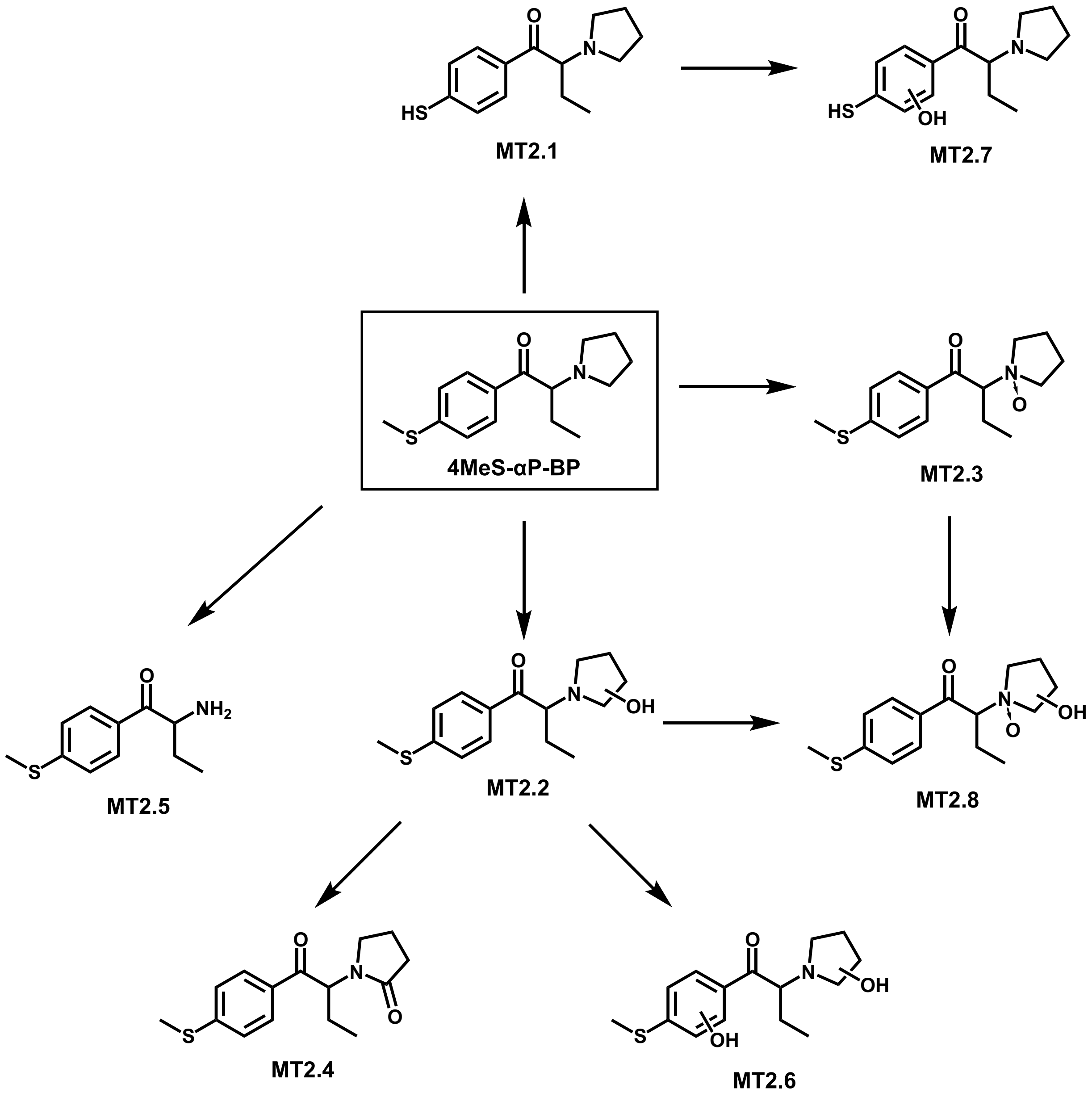

Figure S5 In vitro metabolic pathways of 4MeS-αP-BP in HepaRG, pHLS9 and/or monooxygenases incubations. Metabolite-IDs refer to Table S2.5. →, metabolized to.

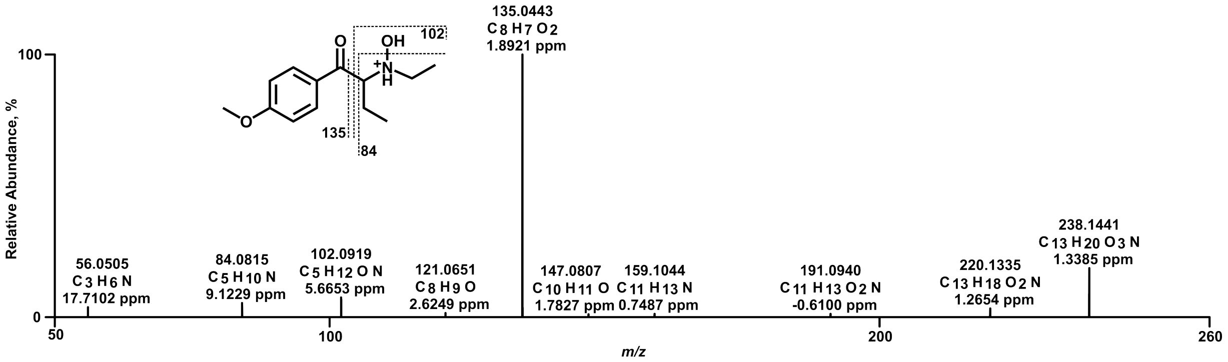

MO1.4 Hydroxylamine formation (*m/z* 238.1438)
RT: 5.50

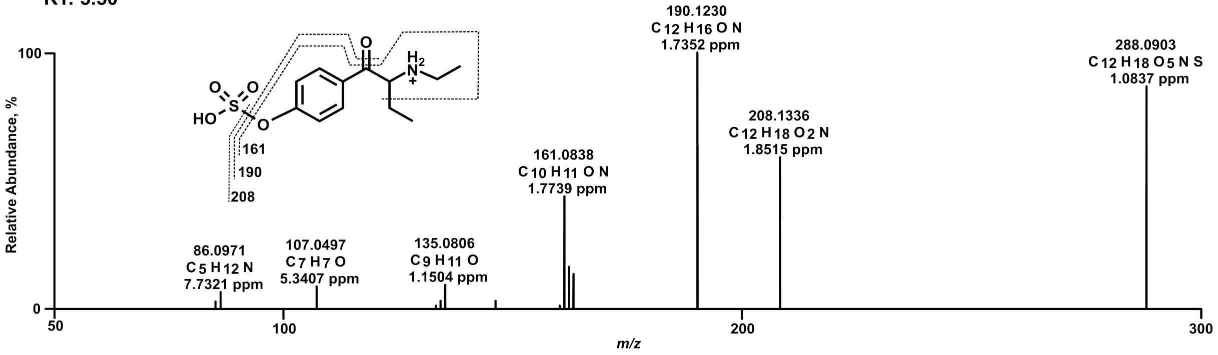

MO1.5 *O*-demethylation + sulfation (*m/z* 288.0900)
RT: 3.91

Figure S6 Additional spectra of 4MeO-NE-BP metabolites, sorted by descending abundance.

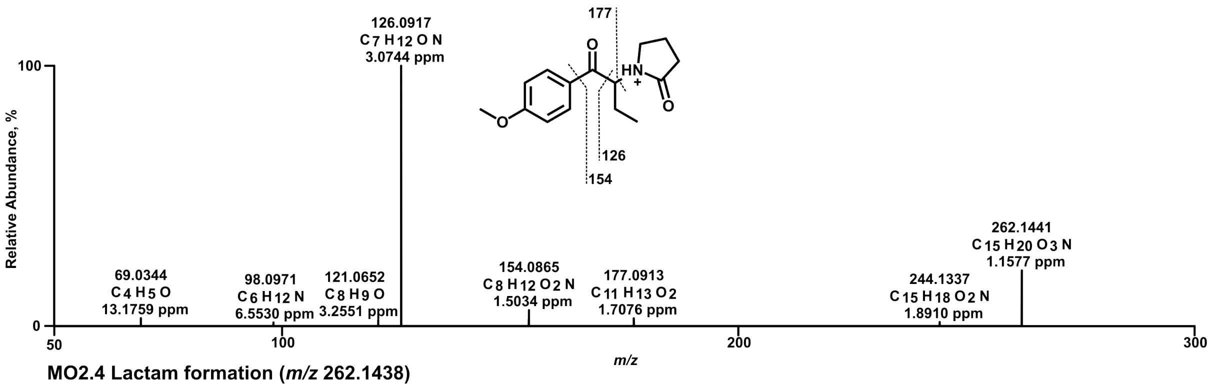

MO2.4 Lactam formation (*m/z* 262.1438)
RT: 6.44 min

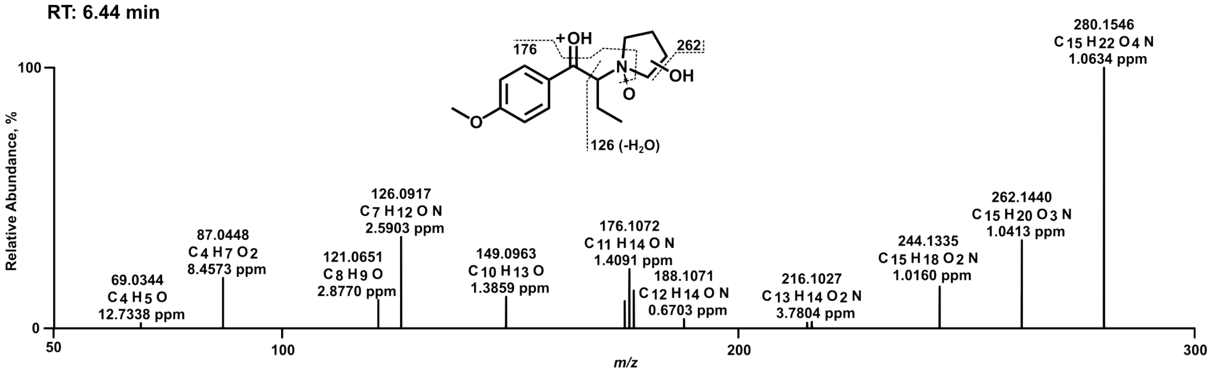

MO2.5 Hydroxylation + *N*-oxidation (*m/z* 280.1543)
RT: 4.98 min

Figure S7 Additional spectra of 4MeO-αP-BP metabolites, sorted by descending abundance.

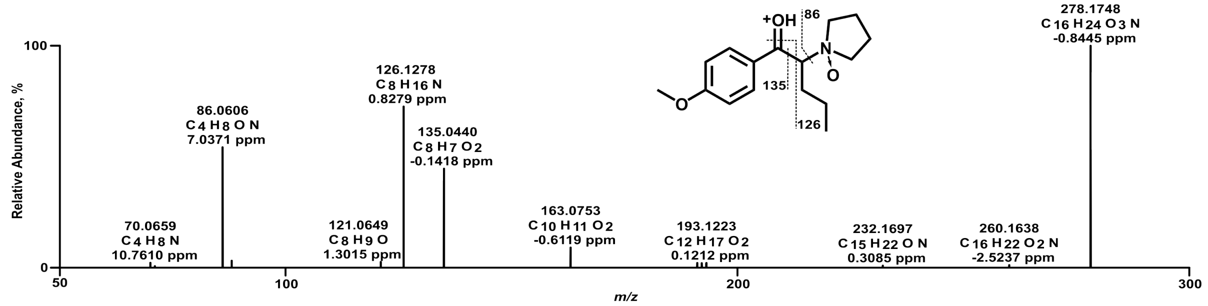

MO3.4 *N*-oxidation (*m/z* 278.1751)
RT: 5.64 min

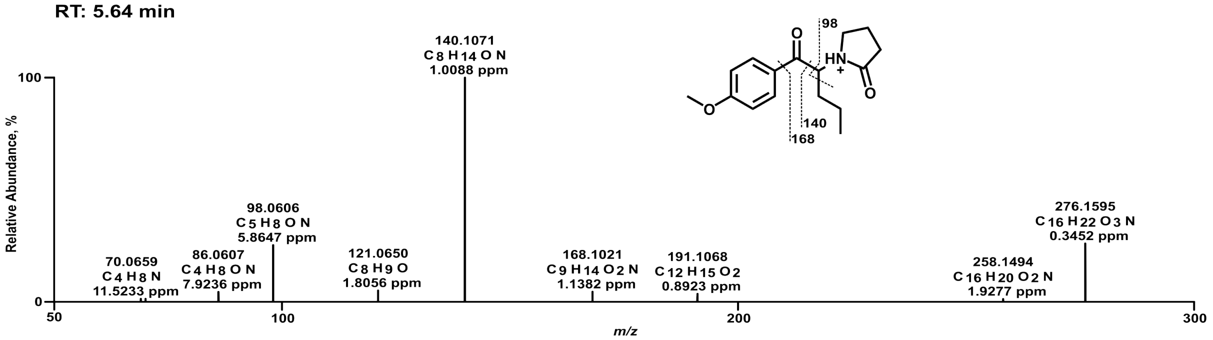

MO3.5 Lactam formation (*m/z* 276.1594)
RT: 6.68 min

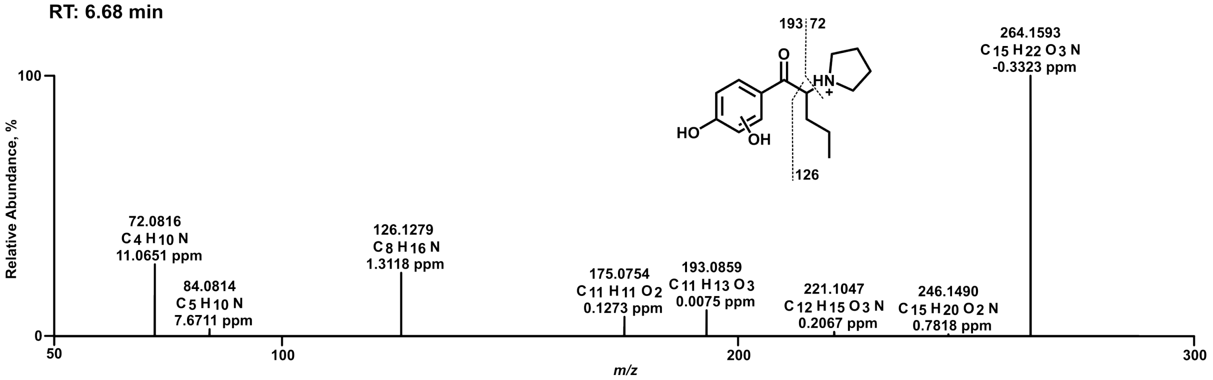

MO3.6 *O*-demethylation + hydroxylation (*m/z* 264.1594)
RT: 4.47 min

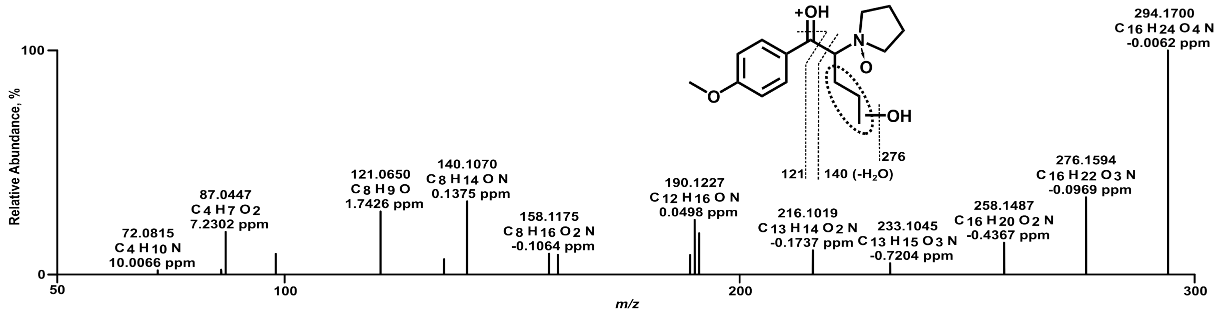

MO3.7 Hydroxylation + *N*-oxidation (*m/z* 294.1700)
RT: 5.24 min

Figure S8 Additional spectra of 4MeO-αP-VP metabolites, sorted by descending abundance.

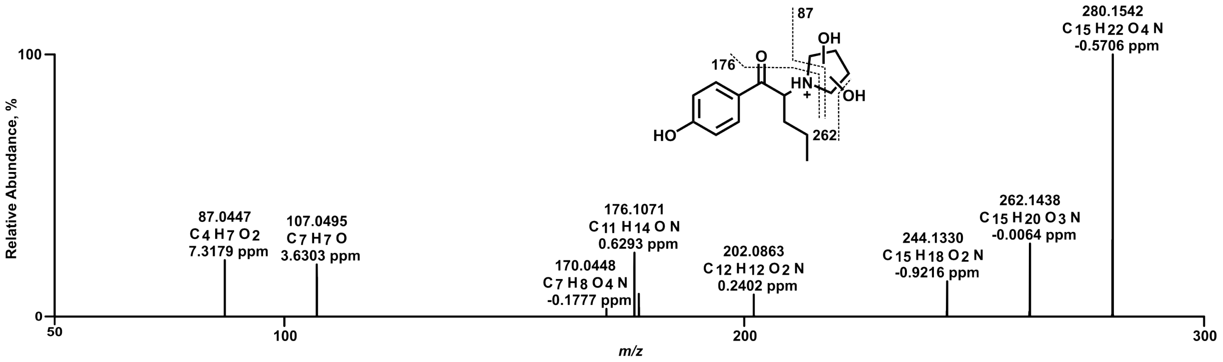

MO3.8 *O*-demethylation + dihydroxylation (*m/z* 280.1543)
RT: 4.66 min

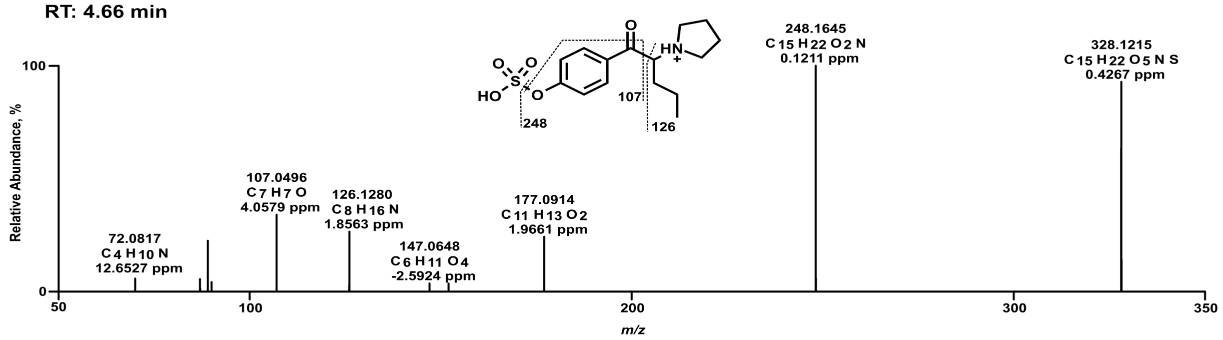

MO3.9 *O*-demethylation + sulfation (*m/z* 328.1213)
RT: 4.68 min

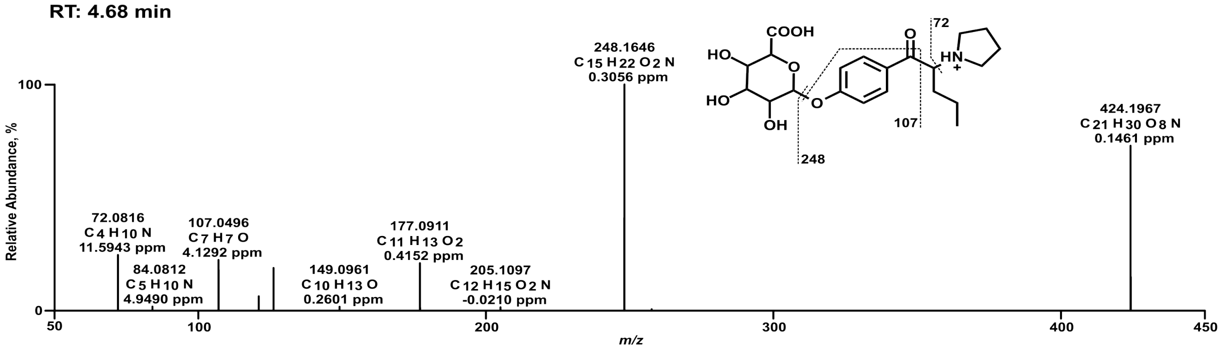

MO3.10 *O*-demethylation + glucuronidation (*m/z* 424.1966)
RT: 3.91 min

Figure S8 Continued.

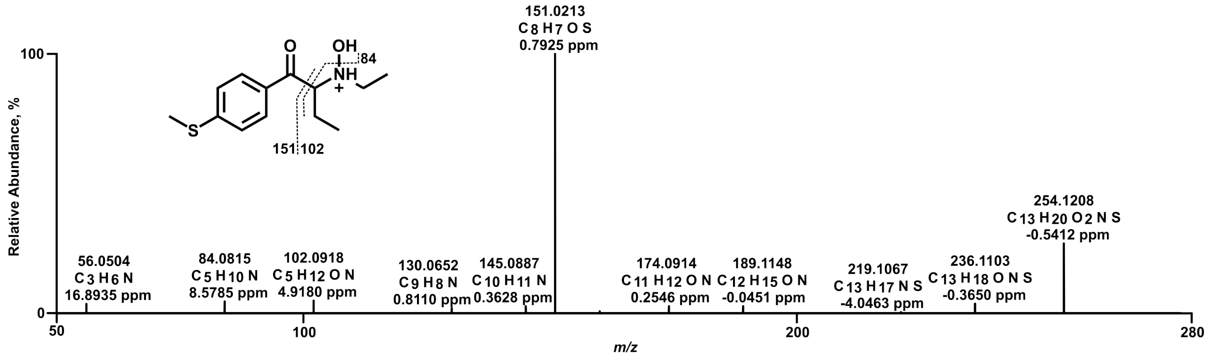

MT1.4 Hydroxylamine formation (*m/z* 254.1209)
RT: 6.23 min

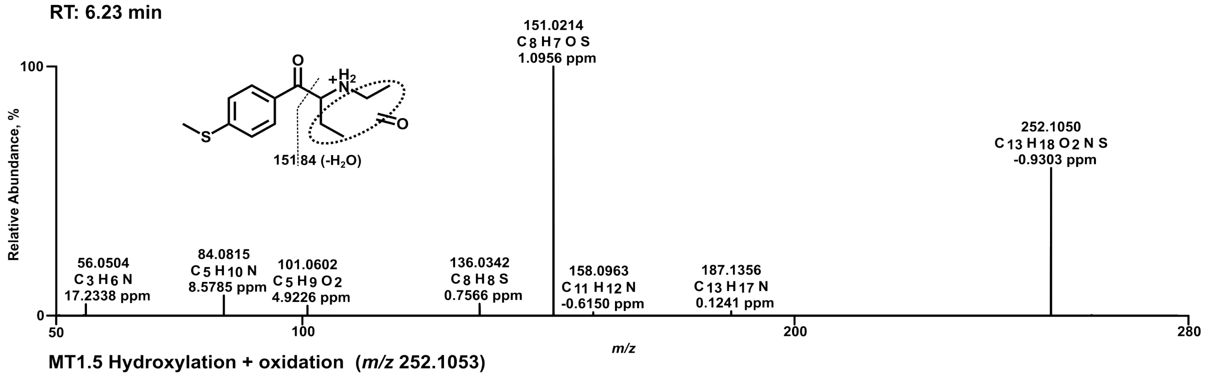

MT1.5 Hydroxylation + oxidation (*m/z* 252.1053)
RT: 6.46 min

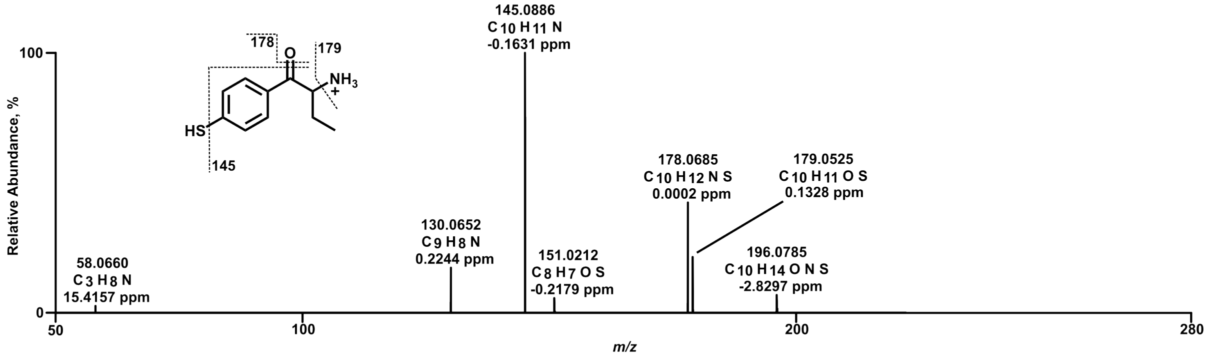

MT1.6 *N*-dealkylation + *S*-demethylation (*m/z* 196.0791)
RT: 4.88 min

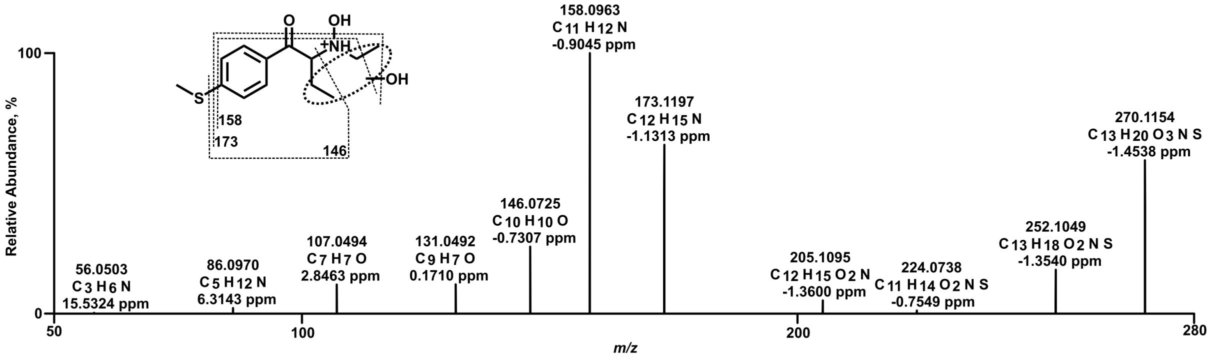

MT1.7 Hydroxylamine formation + hydroxylation (*m/z* 270.1158)
RT: 5.50 min

Figure S9 Additional spectra of 4MeS-NE-BP metabolites, sorted by descending abundance.

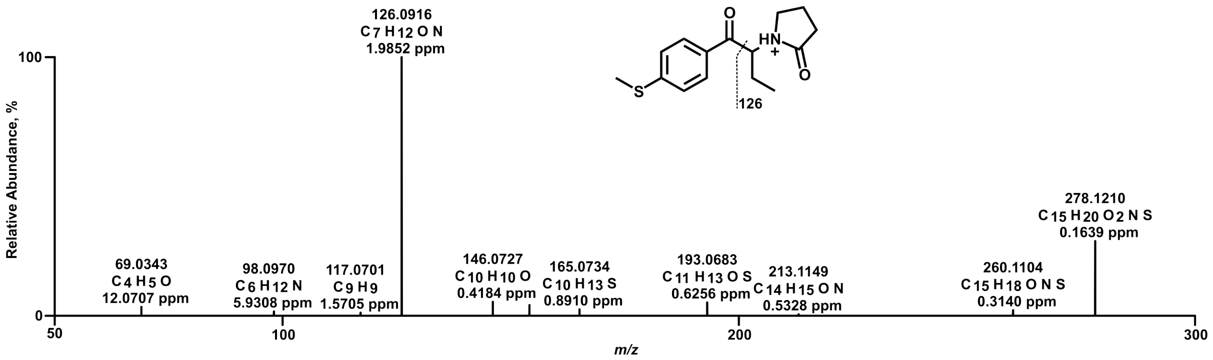

MT2.4 Lactam formation (*m/z* 278.1209)
RT: 6.77 min

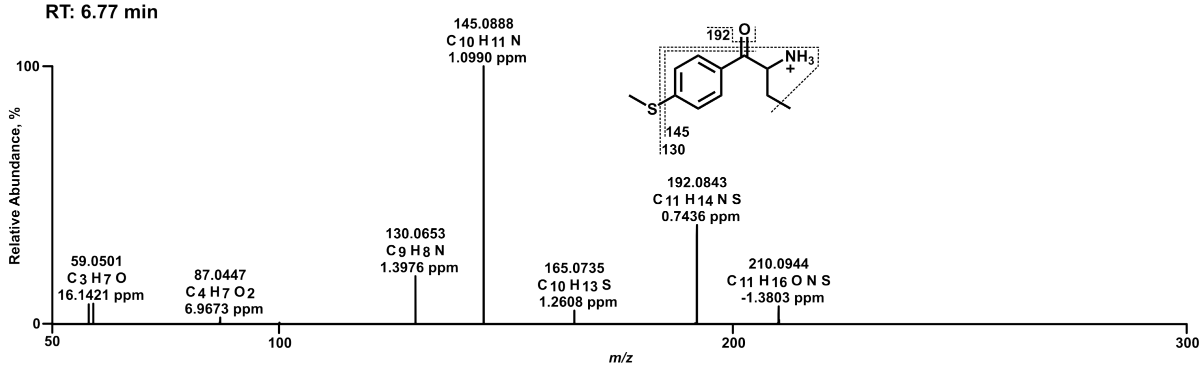

MT2.5 *N*,*N*-bis-dealkylation (*m/z* 210.0947)
RT: 5.22 min

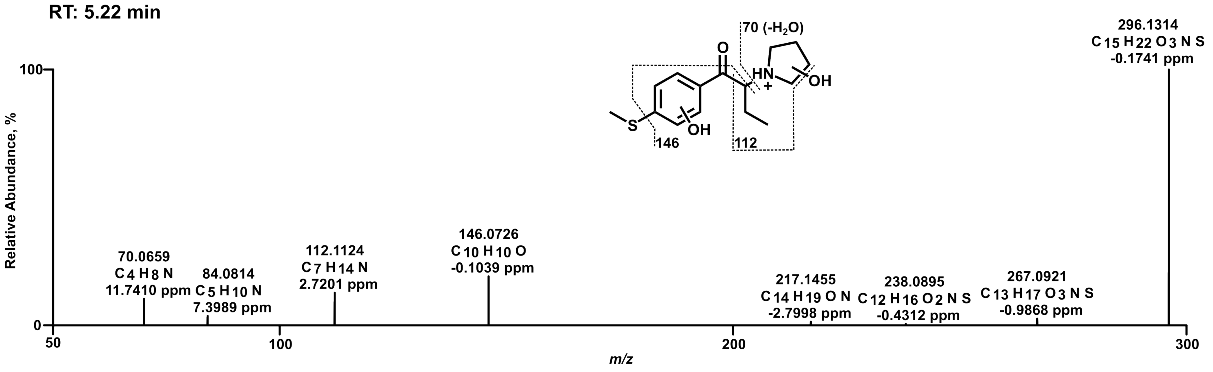

MT2.6 Dihydroxylation (*m/z* 296.1315)
RT: 4.35 min

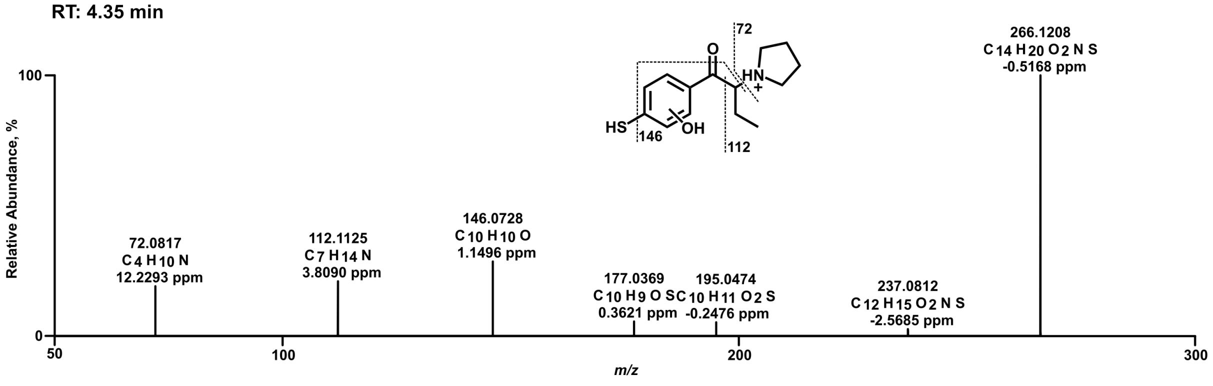

MT2.7 *S*-demethylation + hydroxylation (*m/z* 266.1209)
RT: 5.06 min

Figure S10 Additional spectra of 4MeS-αP-BP metabolites, sorted by descending abundance.

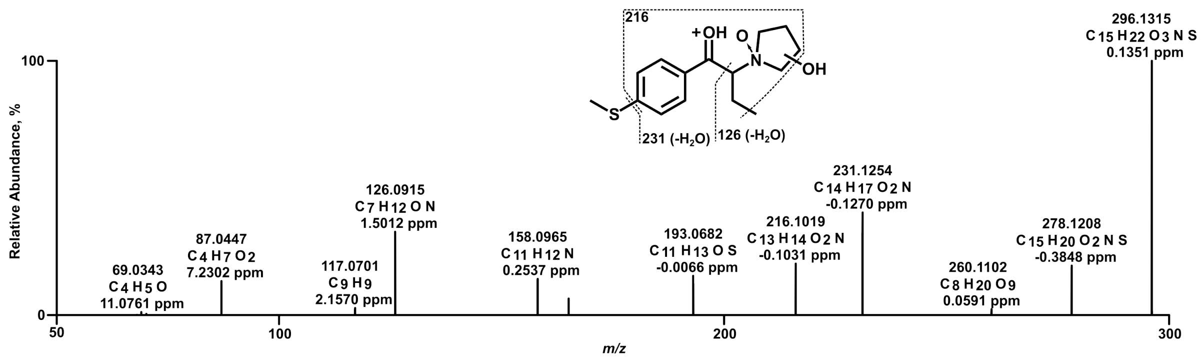

MT2.8 *N*-oxidation + hydroxylation (*m/z* 296.1315)
RT: 5.32 min

Figure S10 Continued.

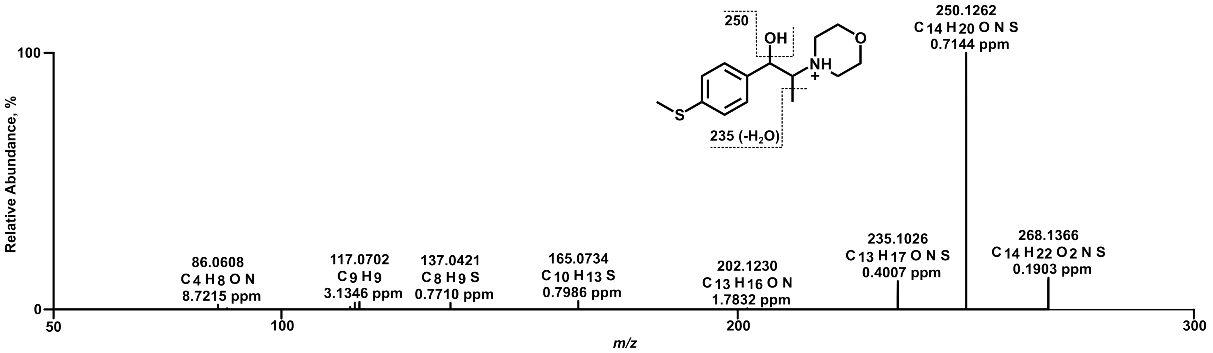

MT3.4 Oxo reduction (*m/z* 268.1366)
RT: 5.00 min

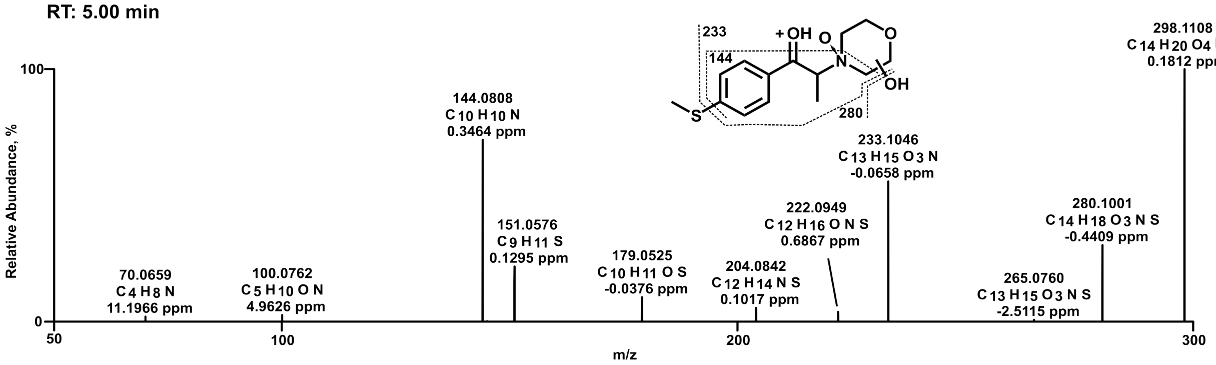

MT3.5 Hydroxylation + *N*-oxidation (*m/z* 298.1108)
RT: 5.13 min

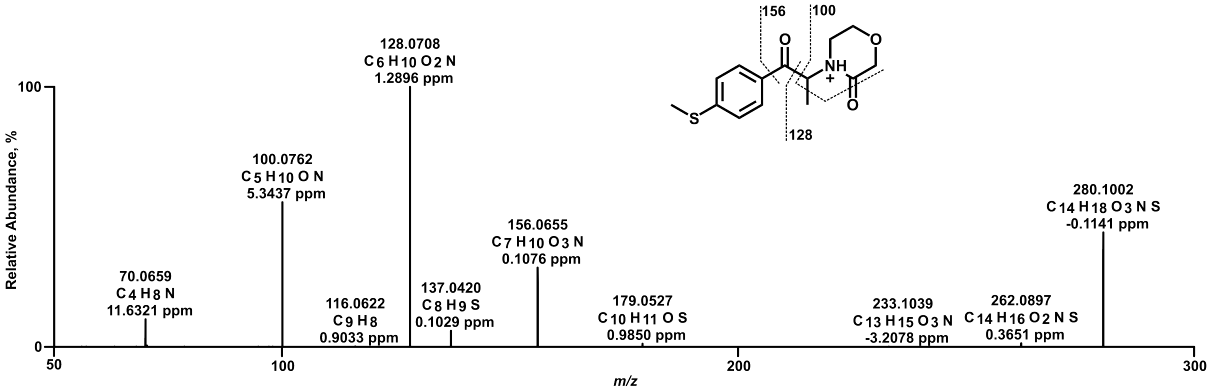

MT3.6 Lactam formation (*m/z* 280.1002)
RT: 6.34 min

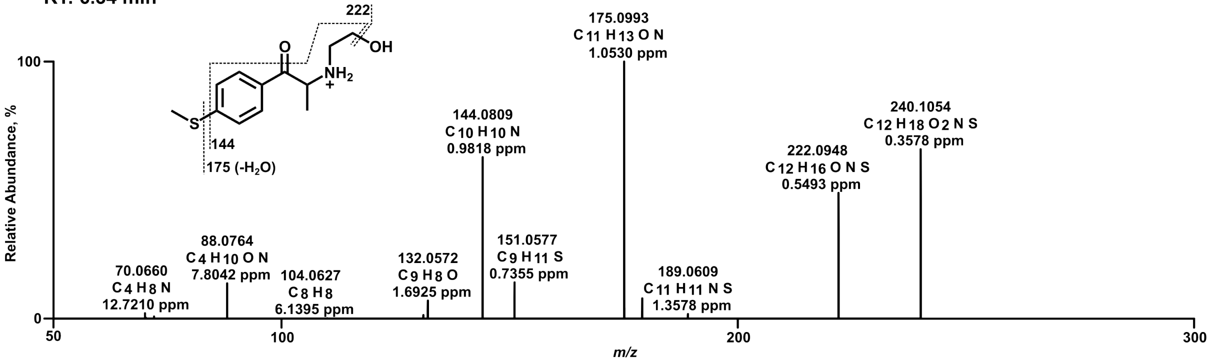

MT3.7 *N*,*O*-bis-dealkylation (*m/z* 240.1053)
RT: 4.93 min

Figure S11 Additional spectra of 4MeS-αMor-PrP metabolites, sorted by descending abundance.

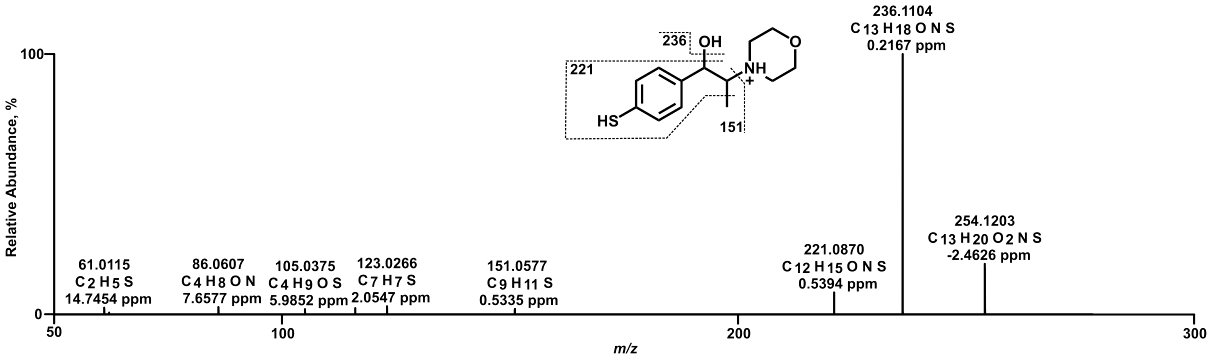

MT3.8 *S*-demethylation + oxo reduction (*m/z* 254.1209)
RT: 4.56 min

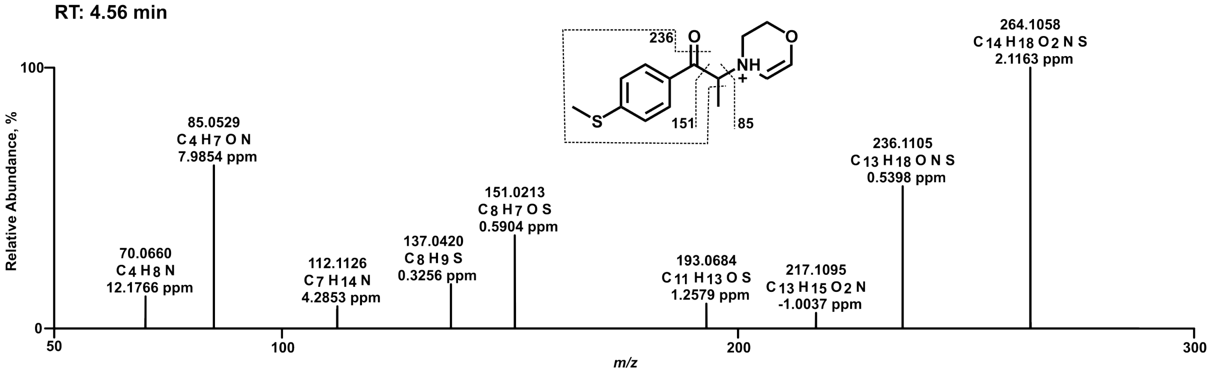

MT3.9 Dehydrogenation (*m/z* 264.1053)
RT: 5.21 min

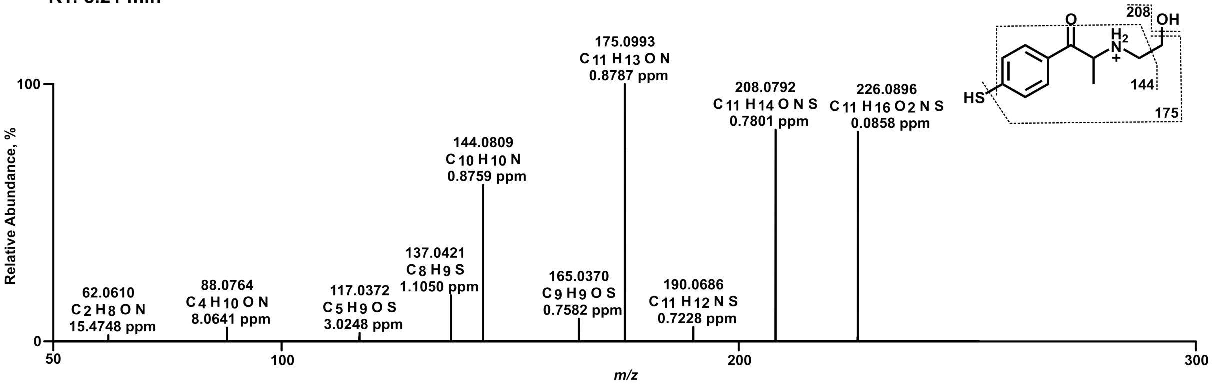

MT3.10 *N*,*O*-dealkylation + *S*-demethylation (*m/z* 226.0896)
RT: 4.40 min

Figure S11 Continued.
